# Flow laws for ice constrained by 70 years of laboratory experiments

**DOI:** 10.1038/s41561-025-01661-z

**Published:** 2025-03-28

**Authors:** Sheng Fan, Ting Wang, David J. Prior, Thomas Breithaupt, Travis F. Hager, David Wallis

**Affiliations:** 1 Department of Geology, University of Otago, Dunedin, New Zealand; 2 Department of Earth Sciences, University of Cambridge, Cambridge, UK; 3 Department of Mathematics and Statistics, University of Otago, Dunedin, New Zealand; 4 Department of Earth and Environmental Science, University of Pennsylvania, Philadelphia, PA USA; 5 Department of Geology, University of Maryland, College Park, MD USA

**Keywords:** Structural geology, Cryospheric science, Climate sciences

## Abstract

Flow laws for ice predict rates of deformation (strain) and are fundamental to modelling glacier and ice-sheet dynamics. Here we apply Bayesian inference to laboratory measurements accumulated over 70 years to constrain flow laws for ice-sheet modelling. At low strains, commonly used flow laws—derived from individual experimental datasets with narrow stress, temperature and grain-size ranges—fail to capture the full complexity of ice behaviour. We show that a multicomponent flow law that sums strain rates from different deformation mechanisms is needed to capture grain-size and temperature sensitivities observed in the full set of experiments. This multicomponent flow law is applicable to natural scenarios where the anisotropy of ice is weak or where the deformation kinematics differ from those that formed the crystallographic preferred orientation, making the ice more viscous. Low-strain flow laws, including this multicomponent flow law, have limited validity at high strain, where viscosity evolves and anisotropy develops, making ice less viscous. A one-component, grain-size insensitive flow law gives a reasonable fit to high-strain experimental data and is better suited to modelling the large-scale flow behaviour of ice sheets.

## Abstract (web summary)

Experimentally constrained flow laws predict ice-sheet strain rates that differ by an order of magnitude from estimates made using previous flow laws, highlighting the need for accurate flow laws in ice-sheet modelling.

## Main

A key source of uncertainty in sea-level projections is understanding how rapidly ice sheets will respond to ongoing climate change. Two key factors determine the velocity of ice flowing from land into the ocean, specifically the internal deformation of the ice and the sliding of ice along the bedrock beneath it, known as basal sliding. Both factors are sensitive to changes in the driving forces that cause inland ice to flow seaward^1^. Floating ice shelves that extend from ice sheets can help to restrain inland ice. This supporting effect is called buttressing. However, ongoing ocean warming is causing rapid thinning and calving at the edges of ice shelves, reducing buttressing forces and increasing stresses that drive inland ice movement, drastically accelerating ice-mass loss, and directly affecting sea levels^2^. To accurately assess how these changes will influence sea level, ice-sheet models must quantify robustly the contributions of both internal deformation and basal sliding to overall ice-flow velocity. In practice, such models represent internal deformation through flow laws that define relationships between driving force (stress) and deformation rate (strain rate). Basal sliding laws are calibrated by comparing observed surface velocities with velocities calculated from the flow laws, with any excess attributed to sliding^3,4^. Thus, forecasts of the contribution of basal sliding to ice-mass loss fundamentally depend on flow laws for internal deformation. Both the form of the flow law and the values of its parameters have a substantial impact on model outcomes^5^. Therefore, robust flow laws are critically important for accurate forecasts of future ice-mass loss.

**Fig. 1:**
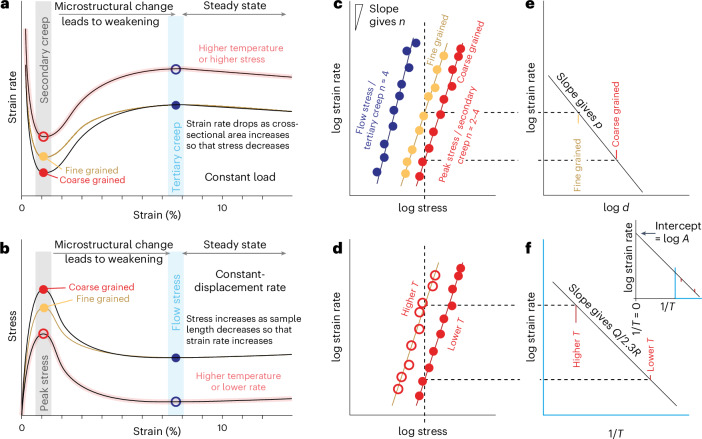
Simplified schematic illustrations to show how flow-law parameters are derived from experimental measurements. **a**,**b**, Mechanical data acquired from individual constant load experiments (**a**) and constant displacement rate experiments (**b**). Each plot shows three experiments (black, yellow and red curves). **c**,**d**, A compilation of data from many experiments plotted as log strain rate versus log stress, focussing on the impact of varying grain size, *d* (**c**) and the effect of temperature, *T* (**d**). Each dot represents a pick of a strain rate-stress pair from an individual experiment as shown in **a** and **b**. **c**,**e**,**f**, Illustrations of how flow-law parameters are calculated from mechanical data, showing the determination of stress exponent, *n* (**c**), grain-size exponent, *p* (**e**), and activation energy, *Q* and scaling constant, *A* (**f**). These schematics are simplified. Real data will be complicated by the operation of more than one mechanism giving different *n*, *p* and *Q* values in different regions of these graphs^27^. Any change in fit of *n*, *p* or *Q* requires a change in *A*.

Flow laws for ice are derived from laboratory experiments^6–8^ (Fig. 1) and inversions of velocity data from remote sensing and the field^9–14^. Experiments offer the advantage of well-defined measurements of stress, strain rate, temperature and material characteristics, such as grain size and crystallographic preferred orientation (CPO). However, flow laws based on experiments must be extrapolated to the lower stresses (typically below 0.1 MPa) and strain rates (typically below 10^−8^ s^−1^) relevant to ice sheets and glaciers. Robust extrapolation from experimental to natural conditions requires flow laws to have both the correct functional form and accurate values of parameters^15^.

Ice-sheet models routinely use the Glen flow law^3,16^, an empirical power-law relationship calibrated for ice that relates strain rate to stress and includes an Arrhenius temperature dependence (Supplementary Information Section 1). However, the Glen flow law does not fit laboratory experiments well (see the next section) and needs substantial modification to match field observations^9^. A revaluation of the experimental constraints on the flow laws for ice is overdue.

Here we reanalyse existing laboratory data obtained over a wide range of conditions using a Bayesian Markov chain Monte Carlo (MCMC) approach that enables us to test constitutive forms and determine flow-law parameters while also accounting for their interdependencies and uncertainties (Methods). We compiled a comprehensive database (Supplementary Table 1) comprising 566 data points from published deformation experiments^6,7,17–38^ along with four additional experiments (Supplementary Information Section 2). Using 305 data points (Supplementary Table 2), we constrain flow laws for low strains (1–2%), relevant to peak stresses or secondary-creep strain rates. At these low strains, the microstructure remains largely unchanged from the isotropic (that is, lacking CPO) starting material. We also use 160 data points (Supplementary Table 3) to constrain a flow law for steady-state flow at higher strains (≳8%), corresponding to flow stresses or tertiary-creep strain rates, at which ice has weakened (Fig. 1) due to microstructural changes that include reduction in grain size and development of anisotropy (strong CPO). Importantly, we assess the resulting flow laws for consistency with the microphysical processes during the deformation of ice.

## Mathematical forms of flow laws

We assume that, under applied stress, polycrystalline materials deform at a bulk strain rate, ${\dot{\varepsilon }}_{{\rm{total}}}$, representing the sum of the strain rates from several components (${\dot{\varepsilon }}_{1},\,{\dot{\varepsilon }}_{2},\,\ldots ,\,{\dot{\varepsilon }}_{m}$), each corresponding to an independent deformation mechanism^39^. We explore flow laws with different numbers of components (${\dot{\varepsilon }}_{1},\,{\dot{\varepsilon }}_{2},\,\ldots ,\,{\dot{\varepsilon }}_{m}$) summed as

1 $${\dot{\varepsilon }}_{{\rm{total}}}={\dot{\varepsilon }}_{1}+{\dot{\varepsilon }}_{2}.\ldots .+{\dot{\varepsilon }}_{m},$$

where *m* indicates the *m*th deformation mechanism. The Glen flow law is based on stress and strain-rate data obtained at strain-rate minima (that is, secondary-creep data; Fig. 1a), and it has the form of a one-component, grain-size insensitive (GSI) constitutive equation^7^, specifically

2 $$\dot{\varepsilon }=A{\sigma }^{n}\exp \left(-\frac{Q}{RT}\right),$$

where $\dot{\varepsilon }$ is strain rate, *σ* is stress, *n* is the stress exponent, *Q* is the activation energy, *R* is the universal gas constant, *T* is absolute temperature and *A* is a scaling constant that encapsulates all unspecified factors (for example, CPO or impurity effects) influencing the deformation.

To account for the relative weakness of ice deformed to high strain (Fig. 1a,b), Durham et al.^20^ derived a similar flow law, based on stresses and strain rates measured at high strain, after mechanical steady state was reached (flow stress; Fig. 1b). The Durham flow law shares the form of the Glen flow law (equation (2)) but with different parameter values (Table 1).

**Table 1:** Summary of parameters for flow laws with different numbers of components

Strain condition	Number of components	Constitutive formula	Flow law	Parameters^a^													
				*n*_GSI_	*Q*_GSI_	log *A*_GSI_	log *A*_GSI,oct._	*n*_GSS1_	*Q*_GSS1_	*p*_GSS1_	log *A*_GSS1_	log *A*_GSS1,oct._	*n*_GSS2_	*Q*_GSS2_	*p*_GSS2_	log *A*_GSS2_	log *A*_GSS2,oct._
Low strain (1–2%)	1	$\dot{\varepsilon }={A}_{{\rm{GSI}}}{\sigma }^{{n}_{{\rm{GSI}}}}\exp \left(-\frac{{Q}_{{\rm{GSI}}}}{{RT}}\right)$	Glen flow law (>263 K)	3	139	20.41	21.24										
			Glen flow law (<263 K)	3	60	4.73	5.56										
			One-component flow law, GSI	3.1	36	0.4	1.26										
		$\dot{\varepsilon }={A}_{{\rm{GSS}}}{\sigma }^{{n}_{{\rm{GSS}}}}\exp \left(-\frac{{Q}_{{\rm{GSS}}}}{RT}\right)$	One-component flow law, GSS					2.8	63	0.8	3.3	4.06					
	2	$$\begin{array}{c}\dot{\varepsilon }={A}_{{\rm{GSI}}}{\sigma }^{{n}_{{\rm{GSI}}}}\exp \left(-\frac{{Q}_{{\rm{GSI}}}}{RT}\right)\\ +{A}_{{\rm{GSS}}}{\sigma }^{{n}_{{\rm{GSS}}}}{d}^{-{p}_{{GSS}}}\exp \left(-\frac{{Q}_{{\rm{GSS}}}}{RT}\right)\end{array}$$	Goldsby–Kohlstedt flow law (>262 K)	4	155	23.84	25	1.8	250	1.4	37.93	38.37					
			Goldsby–Kohlstedt flow law (<262 K)	4	64	5.7	6.85	1.8	70	1.4	2.04	2.48					
			Two-component flow law	3.7	70	6.6	7.66	2.3	63	1.1	2.21	2.8					
	3	$$\begin{array}{c}\dot{\varepsilon }={A}_{{\rm{GSI}}}{\sigma }^{{n}_{{\rm{GSI}}}}\exp \left(-\frac{{Q}_{{\rm{GSI}}}}{RT}\right)\\ +{A}_{{\rm{GSS}}1}{\sigma }^{{n}_{{\rm{GSS}}1}}{d}^{-{p}_{{\rm{GSS}}1}}\exp \left(-\frac{{Q}_{{\rm{GSS}}1}}{RT}\right)\\ +{A}_{{\rm{GSS}}2}{\sigma }^{{n}_{{\rm{GSS}}2}}{d}^{-{p}_{{\rm{GSS}}2}}\exp \left(-\frac{{Q}_{{\rm{GSS}}2}}{RT}\right)\end{array}$$	Three-component flow law with different *n* and *p* for GSS components	3.6	62	5.07	6.1	1.9	52	1.2	−0.93	−0.46	2.5	182	1.9	22.66	23.33
			Three-component flow law with the same *n* and *p* for GSS components	3.7	65	5.53	6.59	2.2	59	1.2	0.43	1	2.2	176	1.2	23.58	24.15
High strain (≳8%)	1	$\dot{\varepsilon }={A}_{{\rm{GSI}}}{\sigma }^{{n}_{{\rm{GSI}}}}\exp \left(-\frac{{Q}_{{\rm{GSI}}}}{RT}\right)$	Durham flow law (>243 K)	4	91	11.8	12.96										
			One-component flow law, GSI	3.5	90	11.9	12.89										

^a^The scaling constant, *A*, has units of MPa^−*n*^ m^*p*^ s^−1^; the activation energy, *Q*, has units of kJ mol^−1^. The logarithms are calculated to base 10. Unless specified, the flow-law parameters are used for predicting axial strain rate with an input of axial stress, temperature and grain size (where applicable). For one-, two- and three-component flow laws, the parameter values represent the median of the posterior distributions. For the Glen flow law, the reported log *A* value is for estimating octahedral shear strain rate. We provide log *A* values converted from an axial flow law to an octahedral flow law or vice versa.

Goldsby and Kohlstedt^8,27^ observed that samples with smaller grain sizes exhibit faster strain rates when normalized to the same stress and temperature, indicating a grain-size sensitive (GSS) deformation mechanism. They proposed a composite flow law with two components that represent dislocation creep (as in equation (2)) and basal dislocation glide limited by grain-boundary sliding (GBS; Table 1). The GBS component includes the average grain diameter, *d*, and a grain-size exponent, *p*, and is expressed as

3 $$\dot{\varepsilon }=A{\sigma }^{n}{d}^{-p}\exp \left(-\frac{Q}{RT}\right).$$

Both the Durham and Goldsby–Kohlstedt flow laws^8,20^ use axial stress and strain rate, where ‘axial’ refers to the stress and strain rate measured along the direction of loading in a uniaxial experimental setup. This convention aligns with laboratory conditions where stress and strain are typically applied and measured along a single principal axis. In contrast, glaciological studies commonly use octahedral shear stress, which is a scalar measure derived from the three principal stresses, and octahedral shear strain rate, a combined scalar measure of deformation rate based on the principal strain rates. These octahedral measures, derived from the full stress and strain-rate tensors, are more broadly applicable to diverse and complex loading conditions encountered in natural ice masses^23,40^. Published parameter values for the Glen flow law are usually based on octahedral values (Supplementary Information Section 1). The conversion from an octahedral to an axial flow law can be achieved via a multiplication of the scaling constant, *A*, by a value dependent on the stress exponent, *n* (Supplementary Information Section 1). Table 1 includes *A* values for flow laws in both axial (*A*_axial_) and octahedral (*A*_oct._) convention, for both easy comparison with experimental data and use in models.

Flow laws calibrated on low-temperature experiments (typically below −10 °C) tend to underestimate strain rates at higher temperatures (typically above −10 °C), probably due to increased premelting at grain boundaries near the melting temperature^41–43^. Some flow laws have different *Q* values for different temperature regimes (for example, Table 1) separated by arbitrary thresholds between −18 °C and −10 °C (refs, ^8,42,43^), but this approach introduces discontinuities into predicted strain rates that are not present in experimental data (Supplementary Information Section 3).

To determine how many mathematical components are needed to represent low-strain experimental data, we derived flow laws with one to four components (Table 1). The one-component flow law is either GSI (*p* = 0; equation (2)) or GSS (equation (3)). In two-component and three-component flow laws, the first component is predefined as GSI, and the rest are GSS. Our two-component flow law mirrors the form of the Goldsby–Kohlstedt flow law. The three-component flow law includes two GSS components representing a single mechanism with temperature-dependent activation energy. In the four-component flow law, with two components defined as GSI and the remaining two components as GSS, one GSI component contributes negligibly (Supplementary Information Section 4). Owing to the lack of grain-size data at high strains, we could only fit a one-component GSI flow law to these data.

**Extended Data Fig. 1:**
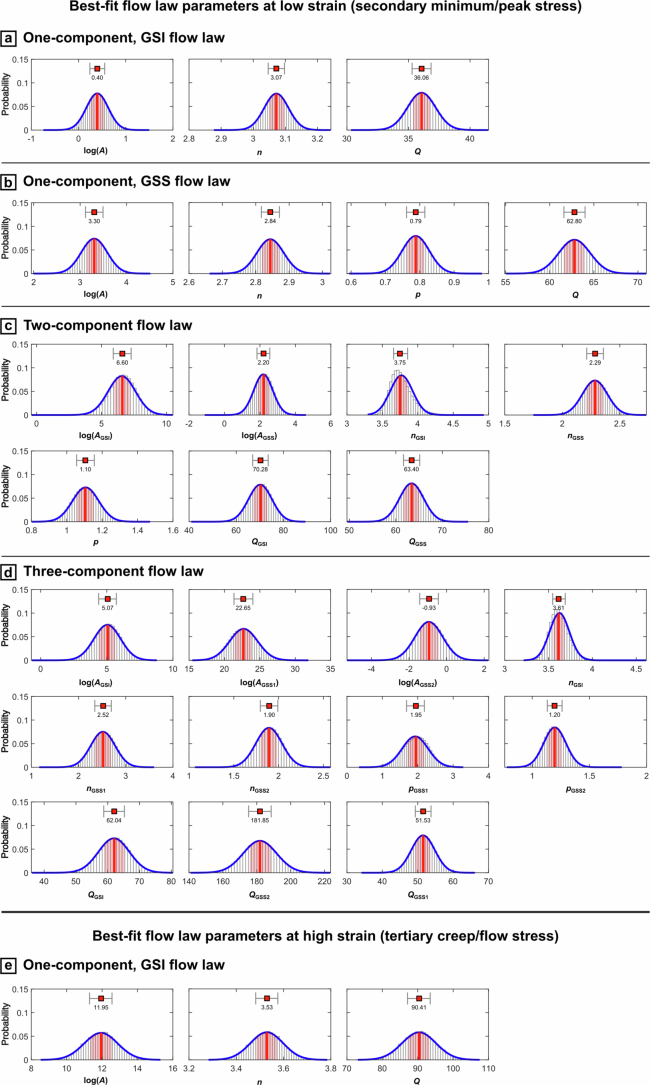
Posterior distributions of parameters for flow laws with axial stress/strain rate convention. **a, b, c**, and **d**, Flow-law parameters at low strain, corresponding to the secondary minimum strain rate (for constant load experiments) or peak stress (for constant displacement rate experiments). **e**, Flow-law parameters at high strain, corresponding to the tertiary-creep strain rate (for constant load experiments) or flow stress (for constant displacement rate experiments). The posterior distribution is depicted as a histogram for each parameter, superimposed with a fitting normal distribution (blue solid curve). The distribution of each parameter in **a**, **b**, **c**, **d**, and **e** is derived from a dataset comprising 598500 samples generated from Bayesian modelling. The interquartile range is emphasised with a light-red shaded area on the histogram and corresponds to the horizontal error bar above. The median is denoted by a pronounced vertical line on the histogram and a red square on the error bar. Quartile values are indicated in grey, with the median highlighted in red. Source data

Bayesian inference combines experimental measurements with prior knowledge to produce posterior distributions that reflect both data and initial assumptions of parameters (Methods). The MCMC method provides a practical tool to sample from complex posterior distributions. Using an iterative process, parameter values are repeatedly sampled from the posterior distribution until a stable set of samples is obtained, that is, the empirical distribution of the samples no longer changes substantially, a state known as convergence. Statistical diagnostics are used to confirm convergence, ensuring the final estimates accurately reflect the most likely parameter values and their uncertainties. Posterior densities and summary statistics, such as the median and credible intervals, can then be obtained for the model parameters from these MCMC samples. We initiated Bayesian inference with prior distributions for *n*, *Q*, *A* and *p* for each flow-law component. Median prior values of *n*, *Q* and *p* are from ref. ^42^. Extended Data Table 1 gives the prior and posterior statistics. Bayesian inference predicts that posteriors of *n*, *Q*, *p* and log *A* tend to follow normal distributions for one-component flow laws. For two-component and three-component flow laws, while most of the posterior distributions appear symmetric, they do not all follow normal distributions (Extended Data Fig. 1).

**Fig. 2:**
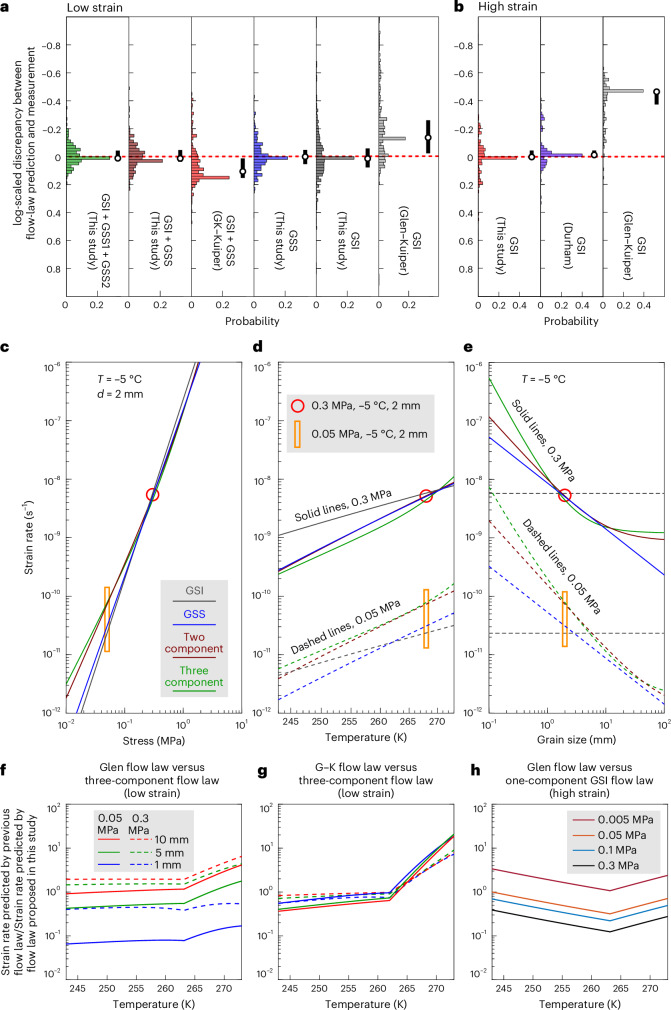
Comparison of the fit of flow laws with experimental data and exploration of the flow laws to natural conditions. **a**,**b**, Distribution of the discrepancy, Δ_*P*−*M*_, plotted on a base 10 logarithmic scale, between the flow-law predictions and experimental measurements (Methods) at low strain (1–2%; **a**) and high strain (≳8%; **b**). The use of the Glen flow law^16^ with calibrated parameters^42^ is shortened as Glen‒Kuiper. The use of the Goldsby-Kohlstedt flow law^8^ with calibrated parameters^42^ is shortened as GK‒Kuiper. The use of the Durham flow law with corresponding parameters^20^ is shortened as Durham. Each histogram in **a** represents data from 305 experiments, while each histogram in **b** represents data from 160 experiments. Values of log Δ_*P*−*M*_ greater than 0 and less than 0 suggest that the flow law predicts strain rates that are faster or slower than those measured, respectively. The black vertical bar represents the interquartile range of log Δ_*P*−*M*_. The white circle represents the median value of log Δ_*P*−*M*_. More complete discrepancy graphs are presented in Extended Data Figs. 2 and 3. **c**–**e**, Predictions of strain rate as a function of stress (**c**), temperature (**d**) and grain size (**e**) using the one-, two- and three-component flow laws constrained by low-strain experimental data. The red circle represents conditions that are commonly used in laboratory experiments. The figure highlights how predictions of flow laws using different numbers of components diverge as conditions deviate from those of the experiments. **f**,**g**, Strain-rate predictions, contrasting the three-component flow law constrained by low-strain experimental data with the published Glen flow law (**f**) and Goldsby–Kohlstedt (G–K) flow law (**g**). **h**, Comparison of strain-rate predictions, contrasting the one-component, GSI law constrained by high-strain experimental data with the Glen flow law. Source data

**Extended Data Fig. 2:**
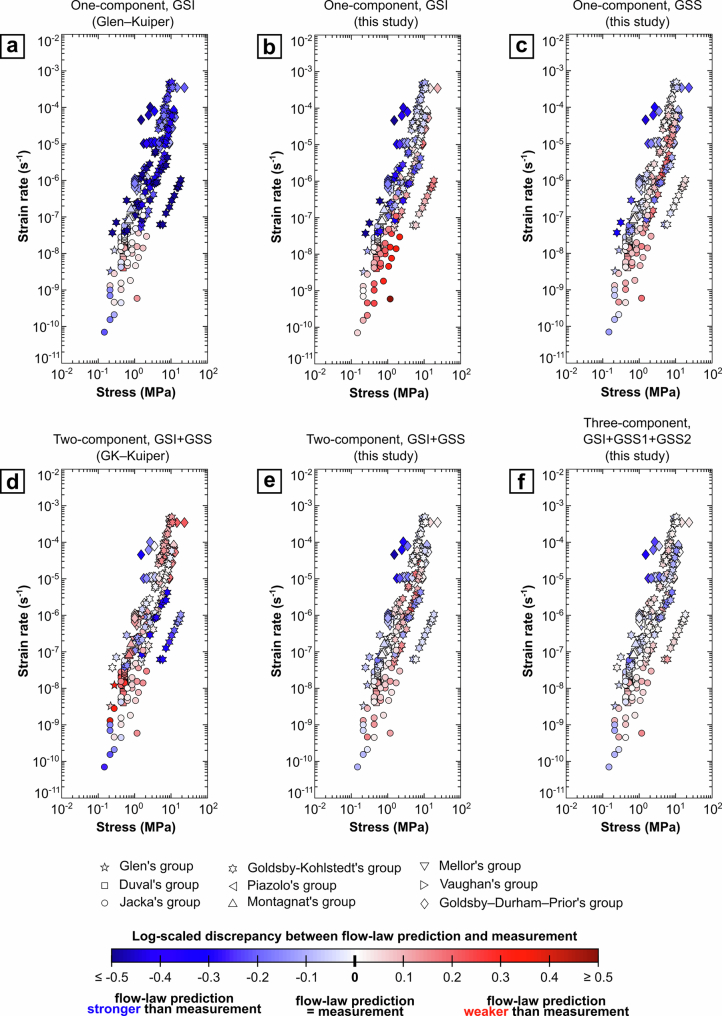
Comparison of flow-law predictions and experimental measurements at low strain. Measured stresses are plotted against measured strain rates with symbols coloured by discrepancy, $\log ({\Delta }_{{\rm{P}}-{\rm{M}}})$, between flow law and measurement (Methods), respectively, at low strain (1–2%). **a**, **b**, and **c**, Comparisons with one-component flow laws: Glen flow law (**a**), GSI flow law (**b**), and GSS flow law (**c**). **d** and **e**, Comparisons with two-component flow laws: Goldsby–Kohlstedt flow law (**d**) and a two-component flow law with parameters evaluated in this study (**e**). **f**, Comparisons with the three-component flow law. Pale shades signify a close agreement between flow-law predictions and measurements, whereas darker shades highlight deviations between the two. Shapes differentiate experiments conducted by various research groups using similar experimental approaches. The five-point star represents Glen’s group^6,7^. The square represents Duval’s group^17^. The circle represents Jacka’s group^21–25,28^. The six-point star represents Goldsby–Kohlstedt’s group^27^. The left-point triangle represents Piazolo’s group^29^. The up-point triangle represents Montagnat’s group^30^. The down-point triangle represents Mellor’s group^18,19^. The right-point triangle represents Vaughan’s group^31^. The diamond represents Goldsby–Prior–Durham’s group^20,32–38^. The use of the Glen flow law^16^ with calibrated parameters^42^ is shortened as Glen‒Kuiper. The use of the Goldsby-Kohlstedt flow law^8^ with calibrated parameters^42^ is shortened as GK‒Kuiper. Source data

**Extended Data Fig. 3:**
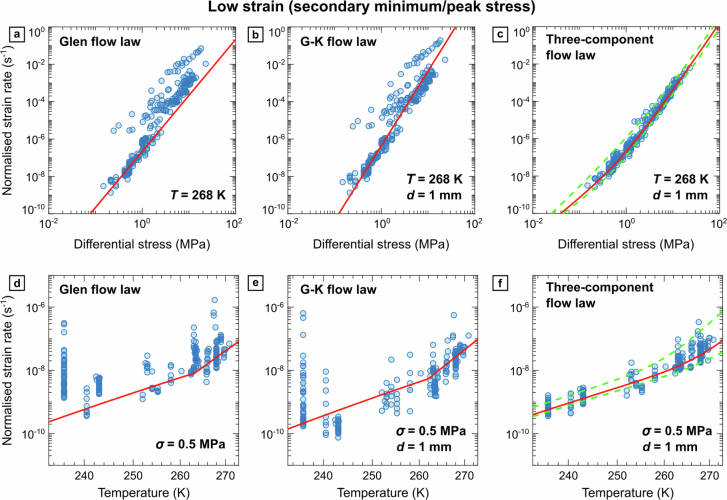
Comparison of normalised measurements (blue circles) with flow-law predictions at low strain. For the Glen flow law and Goldsby-Kohlstedt (shortened as G-K) flow law, the red line represents the prediction using published parameters^42^. In **a–c**, measurements are normalised to temperature of 268 K and grain size of 1 mm (where applicable). In **d–f**, measurements are normalised to stress of 0.5 MPa and grain size of 1 mm (where applicable). In **c** and **f**, the red line represents the prediction based on the median values of the parameters, while the dashed green lines denote the interquartile range of the prediction, constructed using the lower and upper quartiles of the parameter distributions (Table 1), forming an envelope. Source data

**Extended Data Fig. 4:**
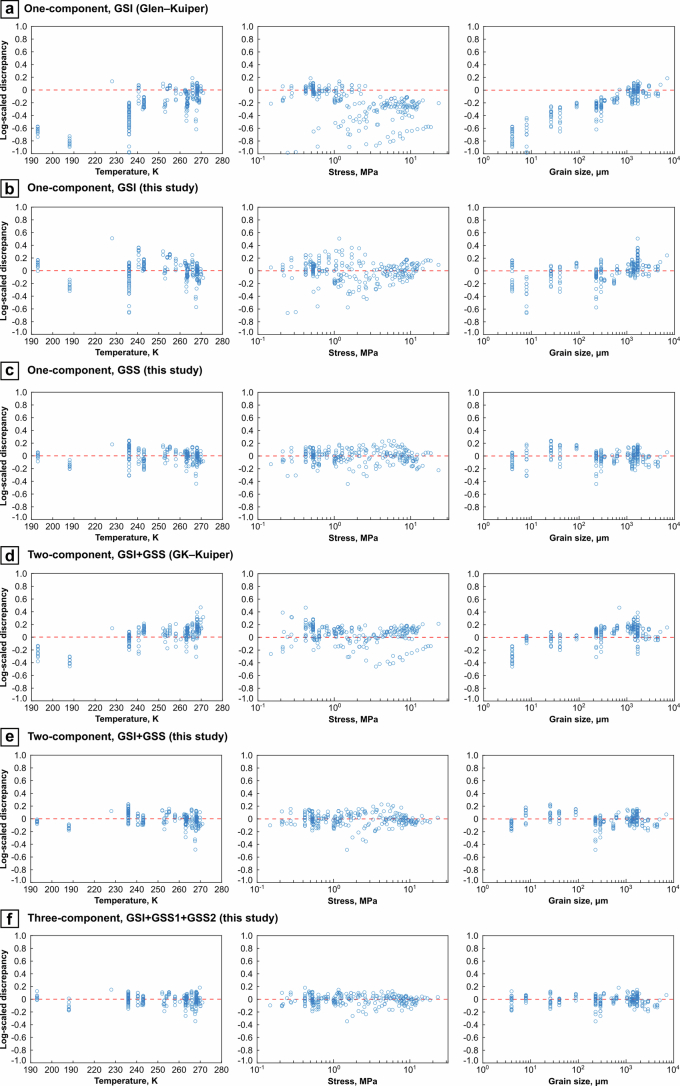
Scattered plots showing the relationship between the discrepancy of the flow-law prediction and the measurements, as functions of temperature, stress, and grain size. **a**, **b**, and **c**, Comparisons with one-component flow laws: Glen flow law (**a**), GSI flow law (**b**), and GSS flow law (**c**). **d** and **e**, Comparisons with two-component flow laws: Goldsby–Kohlstedt flow law (**d**) and a two-component flow law with parameters evaluated in this study (**e**). **f**, Comparisons with the three-component flow law. The red dotted line represents a discrepancy of 1, suggesting that experimental measurement equals to the flow-law prediction. Source data

Our flow laws fit low-strain experimental data better than do the Glen and Goldsby–Kohlstedt flow laws (Fig. 2a and Extended Data Figs. 2 and 3). Only 4% of laboratory data exhibit stresses that differ by a factor greater than 1.5 (approximately half an order of magnitude in strain rate) from the predictions of the three-component flow law, and fewer than 1% differ by a factor greater than 2 (about one order of magnitude in strain rate; Methods). In contrast, 42% and 19% of laboratory data differ by factors greater than 1.5 and 2, respectively, from the predictions of the Glen flow law, while 21% and 5% differ by these respective factors from the predictions of the Goldsby–Kohlstedt flow law. Discrepancies decrease as the number of components increases, as one-component flow laws cannot capture changes in the relative contributions of different deformation mechanisms with deformation conditions. One-component GSI flow laws, such as the Glen flow law, have discrepancies due to grain-size variation (Extended Data Fig. 4). Whilst the Goldsby–Kohlstedt flow law accounts for grain size, it generates discrepancies at higher temperatures because it applies different values of activation energy below and above an imposed temperature threshold (Extended Data Fig. 4). Our two- and three-component flow laws maintain low discrepancies across the full range of experimental variables, highlighting the importance of multicomponent flow laws with improved parameterizations that account for grain-size and temperature effects.

**Extended Data Fig. 5:**
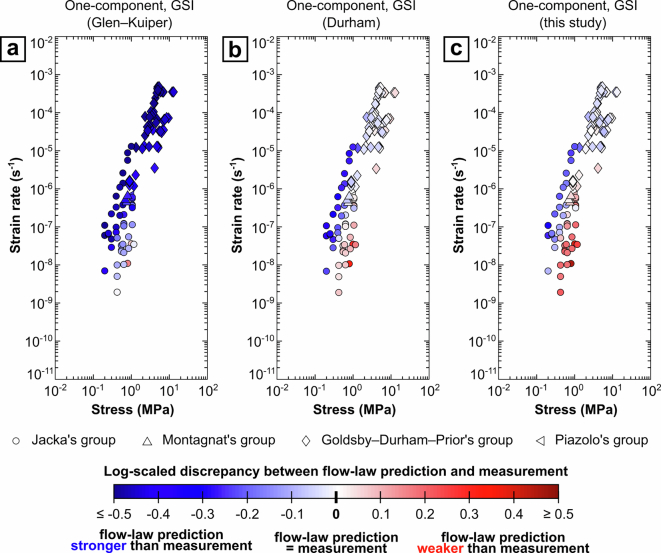
Comparing flow-law predictions with experimental measurements at high strain. Measured stresses are plotted against measured strain rate with symbols coloured by discrepancy, $\log ({\Delta }_{{\rm{P}}-{\rm{M}}})$, between flow law and measurement (Methods), respectively, at high strain (≳8%). **a**, **b**, and **c**, Comparisons with one-component flow laws: Glen flow law (**a**), Durham flow law (**b**) and GSI flow law (**c**). The descriptions correspond directly to Extended Data Fig. 2. Source data

**Extended Data Fig. 6:**
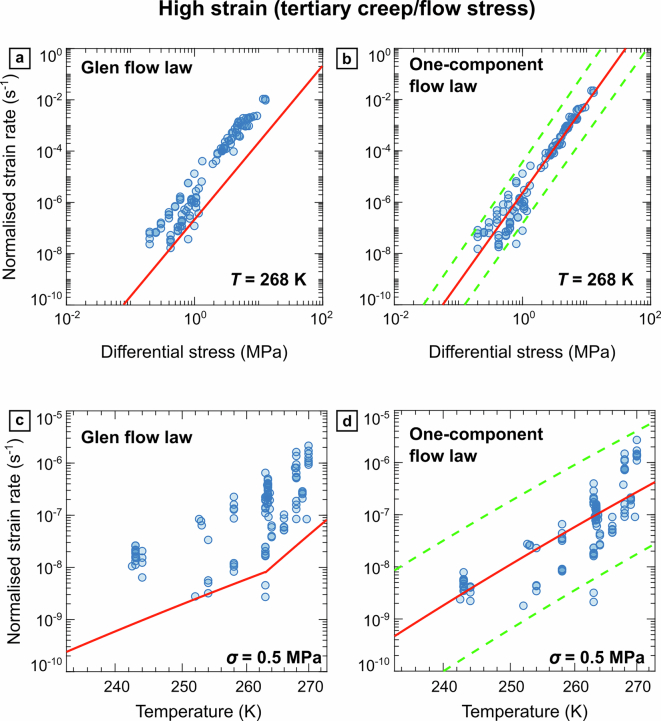
Comparison of normalised measurements (blue circles) with flow-law predictions at high strain. For the Glen flow law, the red line represents the prediction using published parameters^42^. In **a** and **b**, measurements are normalised to temperature of 268 K. In **c** and **d**, measurements are normalised to stress of 0.5 MPa. In **b** and **d**, the red line represents the prediction based on the median values of the parameters, while the dashed green lines denote the interquartile range of the prediction, constructed using the lower and upper quartiles of the parameter distributions (Table 1), forming an envelope. Source data

Our high-strain flow law (one-component GSI) and the Durham flow law perform similarly and fit the high-strain data better than does the Glen flow law (Fig. 2b and Extended Data Figs. 5 and 6). Approximately 20% of laboratory data exhibit stresses that differ by a factor greater than 1.5, and around 1% differ by a factor greater than 2 from the predictions of our high-strain flow law. In comparison, the Glen flow law predicts deviations of approximately 90% and 79%, respectively.

## Physical processes underpinning the deformation of ice

In the two-component and three-component flow laws, the best-fit activation energy, *Q*, for the GSI component is 60–70 kJ mol^−1^ (Table 1), consistent with experimental estimates^44^ and theoretical values based on the volume self-diffusion of oxygen in ice^45^. Dislocation creep in ice involves the movement of oxygen atoms within the crystal lattice, a process governed by self-diffusion. Therefore, the matching activation energies support the widely accepted notion that dislocation creep, a GSI mechanism, is a key deformation mechanism in ice^39^. Our analysis demonstrates that an additional GSS component with nonlinear stress dependence is necessary to fit experimental data. Goldsby and Kohlstedt^8,27^ inferred that the GSS mechanism in their composite flow law involves GBS, which accompanies any deformation where the grain aggregate does not deform homogeneously^46^, such as in diffusion creep and dislocation-accommodated GBS (disGBS)^47^. The GSS components in our two- and three-component flow laws have best-fit stress exponents *n* ≈ 2 and grain-size exponents, *p* ≈ 1–2 (Table 1), consistent with values from disGBS-dominated creep experiments conducted under more limited ranges of conditions^27^. Therefore, we propose that in our two- and three-component flow laws, the GSI component represents dislocation creep, while the GSS components represent disGBS.

**Extended Data Fig. 7:**
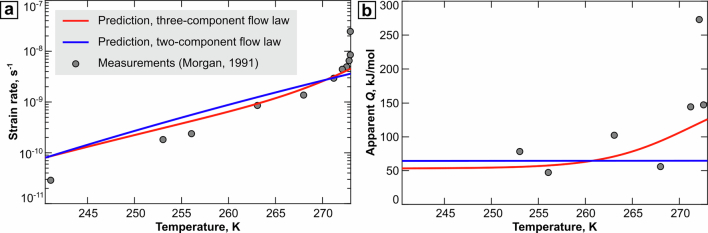
Comparison of two- and three-component flow law predictions with experimental measurements. **a**, Predicted and measured strain rates as a function of temperature, with measured strain rates from Morgan^49^. **b**, Predicted and measured apparent activation energy as a function of temperature, calculated from the measured strain rate-temperature relation. Source data

Our three-component flow law includes two GSS components, raising the question of whether two distinct disGBS components are physically justifiable. The *n* and *p* values for these GSS components are similar and align with theoretical predictions for materials deforming by disGBS^48^. When we constrain the *n* and *p* values of the two GSS components to be identical, the fit remains nearly as good (Supplementary Information Section 5). However, the best-fit *Q* values of the two GSS components are different (Table 1), suggesting that the relative strain-rate contribution from different GSS components depends only on temperature. Thus, the two GSS components may represent a single mechanism with a temperature-dependent activation energy. To test this inference further, we compare these flow laws with data from experiments^49^ that show gradual changes in apparent *Q* near the melting temperature (Extended Data Fig. 7). Our three-component flow law fits the experimental measurements better, predicting an increase in apparent *Q* from ~50 kJ mol^−1^ at −30 °C to 110 kJ mol^−1^ at −3 °C. This observation suggests that the GSS component(s) requires a temperature-dependent *Q*. However, the precise functional form of this temperature dependence remains unknown, and thus we highlight the importance of gaining a better understanding of the physics of premelting than is currently available.

**Extended Data Fig. 8:**
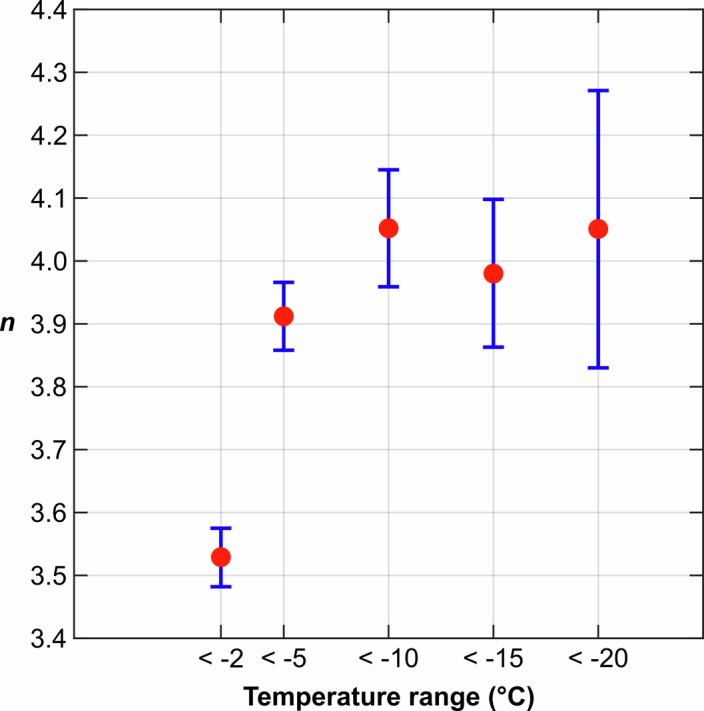
Illustrating the best-fit *n* values for one-component flow law with different temperature thresholds at high strain. Red dots represent the median value, blue whiskers represent the interquartile range. Each interquartile range is based on a dataset comprising 598500 samples generated from Bayesian modelling. Source data

While deformation mechanisms in ice at high strain are probably similar to those at low strain, interactions among the mechanisms become complex due to the development of CPO and changes in grain size with strain^34^. At high strain, a one-component GSI flow law fits the data reasonably well, suggesting that it may be a suitable first approximation. In many materials, the size of recrystallized grains decreases with increasing stress, termed a piezometric relationship^50^, and there is experimental evidence for a piezometric relationship in ice^22^. If grain size is stress-controlled, it may not need to be explicitly included in the flow law, even if GSS mechanisms are active^51^. CPO strength also varies with stress^32,34,36^, possibly eliminating the need to explicitly account for CPO strength in flow laws. Our best-fit flow law has an *n* value of 3.5. If we exclude the highest temperature data (between −2 °C and −5 °C), then the best-fit value of *n* becomes closer to 4 (Extended Data Fig. 8). We speculate that the data collected at higher temperatures could be influenced by mechanisms with Newtonian rheological behaviour at the pressure melting temperature^52,53^.

## Extrapolating flow laws to natural scenarios

At low strain, one- and multicomponent flow laws yield similar strain rates within the narrow ranges of experimental stresses, temperatures and grain sizes. However, when extrapolated to ice-sheet conditions, which involve lower stresses and larger grain sizes, the predictions diverge substantially. At typical glaciological stresses (≤0.1 MPa), multicomponent flow laws predict strain rates an order of magnitude faster than those predicted by one-component flow laws (Fig. 2c, orange box). This divergence increases as temperature (Fig. 2d) and grain size (Fig. 2e) deviate from experimental ranges. Our three-component flow law has the strongest physical basis, encapsulating the observed grain-size and temperature dependencies of ice viscosity with parameter exponents consistent with those predicted by microphysical models of dislocation creep and disGBS (previous section). Therefore, we suggest that the three-component flow law best represents the deformation mechanisms that are active in experiments and ice sheets, and we speculate that it should provide more robust predictions when extrapolated to natural conditions than flow laws with fewer components.

To highlight the importance of well-calibrated flow laws, we compare our flow laws with two previous flow laws that are commonly used in ice-sheet modelling (Fig. 2f‒h). The Glen flow law predicts similar strain rates to those predicted by our low-strain three-component flow law under stress conditions close to experimental settings (for example, dashed lines, Fig. 2f). However, under stress conditions more typical of natural environments (for example, solid lines, Fig. 2f), the Glen flow law predicts strain rates over an order of magnitude slower at a grain size of 1 mm, with this difference decreasing at larger grain size. The Goldsby–Kohlstedt flow law matches our three-component flow law at temperatures below 262 K but predicts strain rates up to an order of magnitude faster above this temperature. The discrepancy between the predictions of our high-strain one-component flow law and those of the Glen flow law is stress dependent, reflecting the different *n* values. The Glen flow law predicts strain rates up to half an order of magnitude faster at stresses below 0.05 MPa, but up to an order of magnitude slower at stresses above 0.05 MPa.

**Fig. 3:**
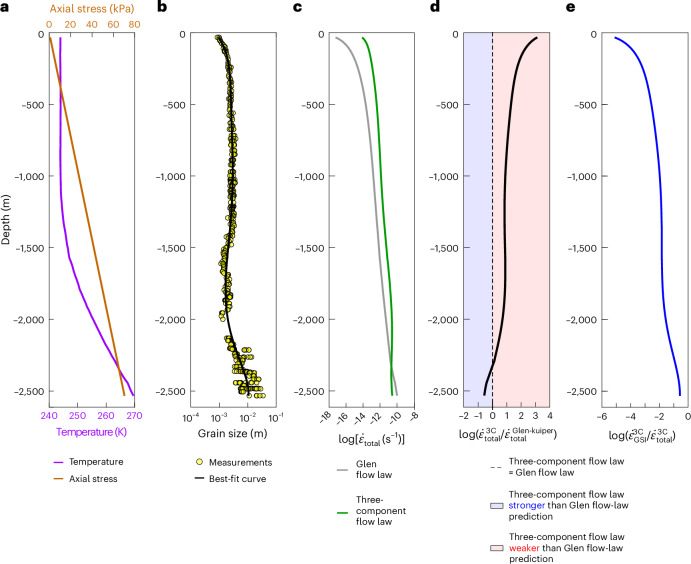
Results of modelling strain rates in the NEEM ice core using different flow laws. **a**, Temperature^58^ and estimated axial stress^56^ as functions of depth. **b**, Grain size as a function of depth, with measurements^57^ represented by yellow dots. The thick solid line indicates the moving-average curve for grain size with depth. **c**, Total strain rates predicted by the Glen flow law and the three-component flow law from this study. **d**, The ratio between total strain rates predicted by the Glen flow law and the three-component flow law from this study. **e**, The ratio between the strain rate contributed by the GSI component and the total strain rate, as predicted by the three-component flow law from this study. In **c** and **d**, ‘3 C’ on the *x* axis denotes the three-component flow law. Source data

To demonstrate differences in the predictions of different flow laws in a natural context, we apply our flow laws to data from the North Greenland Eemian Ice Drilling (NEEM) project. Although the grain sizes are specific to the NEEM ice core, the trend of grain size with depth in the NEEM ice core is similar to that observed in other ice cores, such as those from the Greenland Ice Core Project^54^ and Jarvis Glacier, Alaska^55^. Therefore, using NEEM ice-core measurements as inputs for flow-law predictions provides a general framework for understanding the relationships between grain size, depth and internal deformation in natural glacier ice. We use stress estimates, measured temperatures and grain sizes from ref. ^56^ as shown in Fig. 3a,b (refs. ^57,58^). The Glen flow law predicts strain rates that differ by factors of 0.1‒1,000 compared with our three-component flow law (Fig. 3c,d). In the uppermost 2,300 m, the Glen flow law predicts slower strain rates (Fig. 3c,d), indicating less internal deformation compared with predictions from our three-component flow law. Additionally, our three-component flow law predicts that the contribution of GSI creep to the total strain rate increases with depth, primarily due to increasing differential stress (Fig. 3e).

## Application to ice-sheet modelling

Robust ice-sheet modelling depends on suitable flow laws. Multicomponent flow laws including GSS components give the best fit to low-strain experimental data; the three-component flow law provides the optimal fit. The low-strain flow laws represent the behaviour of isotropic ice that retains its original grain size. Although this situation is rare in nature, low-strain flow laws may be applicable to anisotropic ice when deformation kinematics differ from those that formed the CPO. Examples include glacier borehole closure, where Glen^7^ found that data from borehole closure^40^ match well with predictions based on their low-strain experimental results, as well as flexural deformation near grounding lines^59^, ice transitioning onto an ice shelf^14^ and any scenario with sudden changes in kinematics.

The most appropriate flow law for most ice-sheet models should ideally be derived from high-strain data, which reflect the microstructure (grain size and CPO) established by ongoing deformation kinematics. Unfortunately, the experimental basis for high-strain flow laws remains far more limited than for low-strain regimes. Existing high-strain datasets are sparse and typically lack detailed microstructural characterization, such as measurements of grain size and CPO. Moreover, the narrow ranges of stress and strain rate in current high-strain experiments—often several orders of magnitude faster than those in nature—complicates extrapolation to field conditions. A promising way to resolve this disparity is by obtaining and testing naturally deformed ice that has reached near-steady-state conditions, allowing investigation of relationships between microstructure and mechanical response at more realistic stress and strain rates^60^. Further high-strain experiments, encompassing a broader range of stresses, strain rates and temperatures, are necessary to test and refine the one-component flow law.

Despite these experimental challenges, large-scale ice-sheet models demand practical solutions. Incorporating a multicomponent flow law into continental-scale simulations is hampered by the scarcity of grain-size data across vast ice-sheet regions. Grain size can vary markedly with depth, temperature, impurity content and deformation history^61,62^. Obtaining such data at the spatial resolution needed for continental-scale modelling would require extensive field investigations, ice-core analyses and/or remote-sensing techniques, many of which are resource-intensive and constrained by practical limitations. Encouragingly, our study shows that a one-component, grain-size insensitive flow law provides a reasonable fit to the high-strain experimental data available. This finding points to the possibility of a unified flow law that obviates the need for specifying grain size or CPO, broadening its applicability. Consequently, for models where ice predominantly experiences high strain, the stress exponent, *n*, should be 4 if very high-temperature conditions (*T* > −5 °C) are not considered. However, if the modelling temperature includes *T* > −5 °C, the value of *n* should be 3.5.

## Methods

### Bayesian inference

To determine the best-fit parameters for flow laws, we apply the Bayesian inference, which provides a rigorous framework to estimate the parameters from experimental data, combined with our prior understanding^63^. While the Bayesian framework provides a sound theoretical foundation, in practice, deriving the posterior distributions of parameters analytically can be mathematically challenging, especially for complex models, as is the case in this study, which has up to nine unknown parameters. The MCMC method provides a practical tool to efficiently sample from complex posterior distributions, enabling robust Bayesian inference for intricate models and large datasets^64,65^.

Here we use the MCMC method to generate samples from the posterior distribution of model parameters, implemented in the JAGS software (https://mcmc-jags.sourceforge.io/) and the R2jags package (http://cran.at.r-project.org/web/packages/R2jags) in R. The samplers used in JAGS, which runs via the R2jags package in R, are automatically selected, starting with sampling methods that are efficient (for example, the Gibbs sampler) and ending with the most generic methods (for example, the Metropolis–Hastings algorithm) when needed^66^. After sufficient iterations (~10^6^ in this study), the samples stabilize and converge, yielding a representative posterior distribution of the target parameters (Supplementary Information Section 6). We use three chains, a burn-in of 10^4^ samples and thinning by every 20 samples. Convergence is determined using the potential scale reduction factor $\hat{R}$, which estimates how closely the simulations align with the desired target distribution^67,68^. While $\hat{R} > 1.2$ typically signals non-convergence, we adopt a more stringent rule of $\hat{R} < 1.1$ to ensure robust convergence.

### Prior distributions of flow-law parameters

Extended Data Table 1 presents the constraints on prior distributions of flow-law parameters. For parameters of stress exponent, *n*, grain-size exponent, *p*, and activation energy, *Q*, we use normal distributions centred at mean values, *μ*, adopted from ref. ^42^ and truncate between a minimum and a maximum value as the prior distribution. We choose a modest variance, *σ*^2^, of 0.1 for *n*_GSI_ as *n* = 4 has been relatively well constrained for dislocation creep in ice from previous studies^20,69^. We choose a large variance, *σ*^2^, of 100 for *n* (excluding *n*_GSI_), *p* and *Q*, as they are less constrained by previous experiments, and our confidence in their reported values is accordingly less. We impose a uniform distribution for the material-dependent parameter, *A*, as it is poorly constrained by previous studies.

### Input data

Our database summarizes technical details, such as temperature, experiment type and sample geometry, as well as mechanical data and microstructural statistics, including strain rate, stress, strain and grain size, for 570 experiments (Supplementary Table 1). Where mechanical data are not presented in tabulated form^6,23,24,27,29,30^, we digitized the relevant stresses and strain rates from the figures.

Experiments summarized within the database exhibit variability in their calculation methods for strain rate, strain, stress and grain size. We have addressed these discrepancies by converting and standardizing variables calculated through different methods. For uniaxial compression experiments, we converted the reported engineering strain/strain rate or octahedral shear strain/strain rate to the true axial strain/strain rate following ref. ^34^. Similarly, we converted the reported octahedral shear stress to axial stress following ref. ^36^. For direct-shear experiments, we converted the reported shear strain/strain rate and shear stress to the von Mises strain/strain rate and von Mises stress, respectively, following ref. ^33^. When experiments provide grain-area data, we convert them to area-equivalent diameter^36^.

We have chosen experiments performed at temperatures below −2 °C, given the difficulties in maintaining temperature control near 0 °C. Moreover, we have excluded experiments by ref. ^20^ conducted at temperatures below −40 °C and under exceptionally high stresses (>>10 MPa). These conditions result in a complex mechanical behaviour of ice that is not typically observed in terrestrial ice flow and is associated with a different stress exponent^70^. We also excluded experiments conducted without confining pressure at axial stresses greater than 1.5 MPa due to the potential for sample cracking^18,19^; however, including these data would not substantially change the fitting results (Supplementary Information Section 7). From the remaining experiments, we use the converted strain rate, stress and grain size as inputs for our flow laws.

### Likelihood of experimental measurements

We assume that the strain rate measured from the *i*th experiment, ${\dot{\varepsilon }}_{{\rm{meas}}.}^{i}$, follows a logarithmic normal distribution centred around the expected strain rate, ${\dot{\varepsilon }}_{\exp .}^{i}$, with a variance of 0.1 (equation (4)). This variance corresponds to an experimental error factor of approximately 2 as reported in previous studies^23,30,36^:

4 $$\log {\dot{\varepsilon }}_{{\rm{meas}}.}^{i}{\mathscr{ \approx }}{\mathscr{N}}\left(\log {\dot{\varepsilon }}_{\exp .}^{i},0.1\right).$$

The ${\dot{\varepsilon }}_{\exp .}^{i}$ is the sum of strain rates from different deformation mechanisms,

5 $${\dot{\varepsilon }}_{\exp .}^{i}=\sum _{j}{\dot{\varepsilon }}_{j}\left({\sigma }^{i},{T}^{\,i},{d}^{i}\right),$$

where the subscript *j* denotes deformation mechanism. For a one-component flow law, *j* = 1 and it refers to either GSI or GSS creep. For a two-component flow law, *j* = 1 refers to GSI creep, and *j* = 2 refers to GSS creep. For a three-component flow law, *j* = 1 corresponds to GSI creep, while *j* = 2,3 corresponds to GSS creep. For a four-component flow law, *j* = 1,2 corresponds to GSI creep, while *j* = 3,4 corresponds to GSS creep.

We assume that the average grain size, *d*^*i*^, follows a normal distribution centred at the measured average grain size, ${d}_{{\rm{meas}}.}^{i}$, with a specific variance, ${{s }_{d}^{i}}^{2}$ (equation (6)). For works that do not provide the grain-size distribution, we propose that errors in grain size are proportional to ${d}_{{\rm{meas}}.}^{i}$, and previous works have assumed ${s }_{d}^{i}\approx 0.3{d}_{{\rm{meas}}.}^{i}$ (refs. ^34,71^). For works that provide the grain-size metrics of the upper quartile, ${d}_{{\rm{UQ}}}^{i}$, and lower quartile, ${d}_{{\rm{LQ}}}^{i}$, we impose ${s }_{d}^{i}=({d}_{{\rm{UQ}}}^{i}-{d}_{{\rm{LQ}}}^{i})/1.3$, where 1.3 is the scaling factor between standard deviation and interquartile range:

6 $${d}^{i}{\mathscr{ \approx }}{\mathscr{N}}\left({d}_{{\rm{meas}}.}^{i},{{s }_{d}^{i}}^{2}\right).$$

We assume that the average temperature, *T*^*i*^, follows a normal distribution centred at the measured average temperature, ${T}_{{\rm{meas}}.}^{\,i}$, with a specific variance, ${{s }_{T}^{i}}^{2}$,

7 $${T}^{i}{\mathscr{ \approx }}{\mathscr{N}}\left({T}_{{\rm{meas}}.}^{\,i},{{s }_{T}^{\,i}}^{2}\right).$$

For works that did not provide uncertainties on temperatures, we impose ${s }_{T}^{i}=0.5\,{{\rm{K}}}$, similar to previous studies^32,34,35^. For works that provide the temperature metrics of upper quartile, ${T}_{{\rm{UQ}}}^{i}$, and lower quartile, ${T}_{{\rm{LQ}}}^{\,i}$, we impose ${s }_{T}^{i}=({T}_{{\rm{UQ}}}^{\,i}-{T}_{{\rm{LQ}}}^{\,i})/1.3$.

### Discrepancy between flow-law predictions and experimental measurements

The R2jags package provides a deviance information criterion^72^ as a measure for comparing the performance of different flow laws. The deviance information criterion not only evaluates the goodness of fit but also considers flow-law complexity. A lower deviance information criterion value suggests a better balance between a good fit to the data and flow-law simplicity.

To assess the goodness of fit for each experiment, we determine the discrepancy between flow-law predictions and experimental measurements, Δ_*P*−*M*_. For experiments at constant strain rate, we estimate the stress, *σ*_predict_, using measured average strain rate, temperature and/or grain size coupled with the median values from the posterior distributions of the flow-law parameters (Table 1). The values of Δ_*P*−*M*_ for these experiments are derived by comparing *σ*_predict_ with the measured average stress, *σ*_measure_, given by

8 $${\Delta }_{P-M}=\frac{{\sigma }_{{\rm{measure}}}}{{\sigma }_{{\rm{predict}}}}.$$

For constant load/stress experiments, we estimate the strain rate, ${\dot{\varepsilon }}_{{\rm{predict}}}$, using measured average stress, temperature and/or grain size coupled with the median values from the posterior distributions of the flow-law parameters (Table 1). The values of Δ_*P*−*M*_ for these experiments are derived by comparing ${\dot{\varepsilon }}_{{\rm{predict}}}$ with the measured average strain rate, ${\dot{\varepsilon }}_{{\rm{measure}}}$, given by

9 $${\Delta }_{P-M}={\left(\frac{{\dot{\varepsilon }}_{{\rm{predict}}}}{{\dot{\varepsilon }}_{{\rm{measure}}}}\right)}^{\frac{1}{{\log }^{\prime} {\dot{\varepsilon }}_{{\rm{predict}}}}},$$

where ${\log }^{\prime} {\dot{\varepsilon }}_{{\rm{predict}}}$ denotes the slope of the tangent line to the curve of the flow-law prediction at $\log {\dot{\varepsilon }}_{{\rm{predict}}}$, corresponding to log *σ*_measure_. The logarithms are calculated to base 10.

If flow-law predictions are close to experimental measurements, then log Δ_*P*−*M*_ ≈ 0. A non-zero log Δ_*P*−*M*_ indicates a divergence between flow-law predictions and experimental measurements. Specifically, log Δ_*P*−*M*_ is positive when the flow law predicts a weaker mechanical behaviour and negative when it predicts a stronger mechanical behaviour compared with measurements. We need this approach to compare constant load and constant displacement rate experiments, because strain rate is related to stress through the stress exponent, *n*. For example, the ratio of measured and predicted stress of 2 is equivalent to a ratio of 8 (for *n* = 3) or 16 (for *n* = 4) of measured and predicted strain rate.

## Online content

Any methods, additional references, Nature Portfolio reporting summaries, source data, extended data, supplementary information, acknowledgements, peer review information; details of author contributions and competing interests; and statements of data and code availability are available at 10.1038/s41561-025-01661-z.

## Supplementary information

Supplementary Information Supplementary Sections 1–7, Figs. 1–11 and Tables 1–3.

Supplementary Tables 1–5 Supplementary Table 1: Master database that summarizes technical details, such as temperature, experiment type and sample geometry, as well as mechanical data and microstructural statistics, including strain rate, stress, strain and grain size, for experiments reported in the past 70 years. Supplementary Table 2: Input data used for constraining flow laws at low strain (1–2%). These data exclude data points from high-stress (>1.5 MPa) experiments that use an unconfined medium. Supplementary Table 3: Raw data points used for constraining flow laws at high strain (≳8%). These data exclude data points from high-stress (>1.5 MPa) experiments that use an unconfined medium. Supplementary Table 4: Input data used for constraining flow laws at low strain (1–2%). These data include data points from high-stress (>1.5 MPa) experiments that use an unconfined medium. Supplementary Table 5: Raw data points used for constraining flow laws at high strain (≳8%). These data include data points from high-stress (>1.5 MPa) experiments that use an unconfined medium.

Supplementary Data 1 Raw outputs from the Bayesian modelling.

Supplementary Data 2 Digitized data for published experiments and temperature measurements as a function of depth for the NEEM ice core.

Supplementary Code 1 R code that includes comprehensive markdown notes detailing the Bayesian modelling process.

## Source data

Source Data Fig. 2 Statistical source data.

Source Data Fig. 3 Statistical source data.

Source Data Extended Data Fig. 1 Statistical source data.

Source Data Extended Data Fig. 2 Statistical source data.

Source Data Extended Data Fig. 3 Statistical source data.

Source Data Extended Data Fig. 4 Statistical source data.

Source Data Extended Data Fig. 5 Statistical source data.

Source Data Extended Data Fig. 6 Statistical source data.

Source Data Extended Data Fig. 7 Statistical source data.

Source Data Extended Data Fig. 8 Statistical source data.

## Figures and Tables

**Extended Data Table 1:**
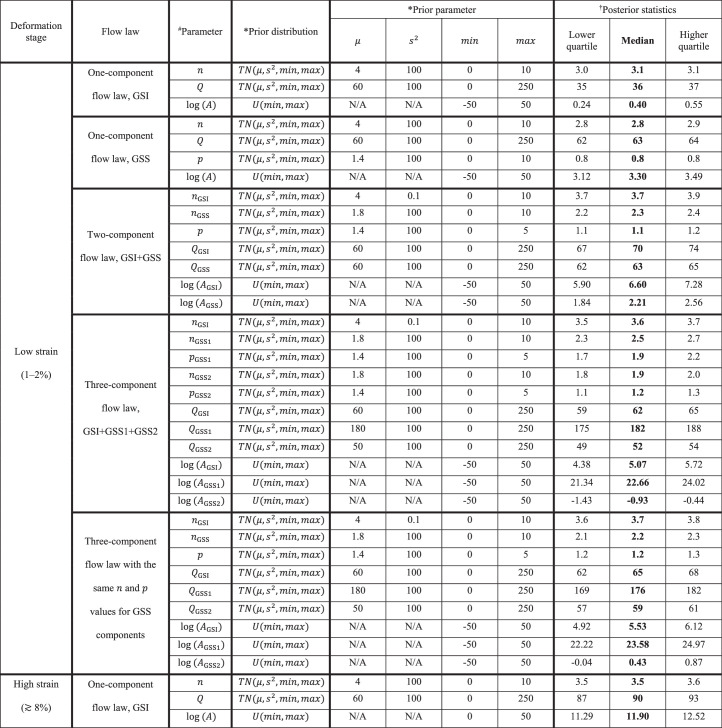
Prior and posterior statistics for parameters of the flow laws

^#^ The scaling constant, $A$, has units of ${{\rm{MPa}}}^{-n}{{\rm{m}}}^{p}{{\rm{s}}}^{-1}$; the activation energy, $Q$, has units of ${\rm{kJ}}{{\rm{mol}}}^{-1}$. The logarithms are calculated to base 10. The flow law parameters are used for predicting axial strain rate with an input of axial stress, temperature, and grain size.

^*^ ${TN}(\mu ,{s }^{2},\min ,\max )$ represents a normal distribution truncated between specified minimum and maximum bounds, with mean, $\mu$, and variance, ${s }^{2}$. $U(\min ,\max )$ represents a uniform distribution where all values between the specified minimum and maximum bounds are equally likely.

^†^ The median, lower quartile, and upper quartile values of each parameter are derived from the posterior distributions obtained through Bayesian statistical modelling.

## Data Availability

Grain-size measurements for the NEEM ice core are available via PANGAEA at 10.1594/PANGAEA.83805 (ref. ^57^). Input data used for the Bayesian modelling are available in Supplementary Tables 1–5. Raw outputs from the Bayesian modelling are provided in Supplementary Data 1. We digitized stress and strain rate data from refs. ^6,23,24,27,29,30^ from figures for experiments where mechanical data were not presented in tabulated form. Temperature measurements as a function of depth for the NEEM ice core were digitized from ref. ^58^. The digitized files are provided in Supplementary Data 2. All the Supplementary Data and source files for Figs. 2 and 3 and Extended Data Figs. 1–8 are publicly available via figshare at 10.6084/m9.figshare.26381212 (ref. ^73^). Source data are provided with this paper.

## Code availability

The R code, which includes comprehensive markdown notes detailing the modelling process, is available in Supplementary Code 1, and is publicly available via figshare at 10.6084/m9.figshare.26381212 (ref. ^73^), via GitHub at https://github.com/ShengFanGeology/BayesianIceFlow and via Code Ocean at 10.24433/CO.6852095.v1 (ref. ^74^).
