# Supplementary material for: Flow laws for ice constrained by 70 years of laboratory experiments
Source: Nat Geosci. 2025 Mar 28;18(4):296–304. doi: 10.1038/s41561-025-01661-z (PMC11981940; doi:10.1038/s41561-025-01661-z)
Supplement: Supplementary file 1 — Supplementary Sections 1–7, Figs. 1–11 and Tables 1–3. [file 41561_2025_1661_MOESM1_ESM.pdf]

nature geoscience

Supplementary information

https://doi.org/10.1038/s41561-025-01661-z

Flow laws for ice constrained by 70 years of laboratory experiments

In the format provided by the authors and unedited

Table of Contents

1. The Glen flow law 2

1.1 Basis of the Glen flow law in the literature 2

Table S1 3

Table S2 5

Figure S1 6

1.2 Axial versus octahedral flow laws 6

2. Mechanical details of additional experiments 9

Figure S2 10

3. Previous flow laws give discontinuous predictions at arbitrary threshold temperatures 11

Figure S3 12

4. Contribution of different components for the four-component flow law 13

Figure S4 14

Figure S5 15

Figure S6 16

Figure S7 17

5. Comparison of posterior distributions of parameters for three-component flow laws 18

Figure S8 18

Figure S9 19

6. MCMC convergence diagnostics 20

Figure S10 21

Figure S11 21

7. Bayesian-inference results with inputs containing data from high-stress (>1.5 MPa) experiments that use an unconfined medium 21

Table S3 22

References 24

1. The Glen flow law

In this section, we outline the basis in the literature of the Glen flow law. We also address a specific issue of whether flow laws are defined using axial or octahedral stresses and strain rates. This is important, as some published flow laws do not state explicitly whether the flow law parameters relate to axial or octahedral stresses and strain rates. We resolve this issue for published flow laws.

1.1 Basis of the Glen flow law in the literature

The origins of the Glen flow law are difficult to trace. Glen uses his ground-breaking experimental work (Glen, 1952, 1953, 1955) to demonstrate a good fit of the power-law relationship between strain rate and stress and an Arrhenius relationship to describe the temperature dependence. However, the Glen flow law, as used in the literature, is not a flow law derived by Glen from his experiments. Key data that provide a basis for commonly used Glen flow-law relations with a stress exponent, n, of 3, are summarised in Table 3 of Budd & Jacka (1989) and many modelling papers (e.g., McCormack et al., 2022; Treverrow et al., 2015) use these data as the basis of a Glen flow law. There are some typographic errors in Table 3 of Budd & Jacka (1989), and we have corrected (bold values) these errors by an order of magnitude to match values plotted in Fig. 7 of Budd & Jacka (1989). The corrected data are shown in Table S1.

Table S1. Strain-rate data commonly used as the basis of the Glen flow law, as a function of temperature.

T (°C)	Budd & Jacka (1989)				Paterson (1994)	Cuffey & Paterson (2010)
	Octahedral minimum stain rate (s^{-1}) at octahedral shear stress = 0.2 MPa	Octahedral minimum stain rate (s^{-1}) at octahedral shear stress = 0.4 MPa	Averaged A_T ($MPa^{-3}s^{-1}$)	Error (%)	A_T ($MPa^{-3}s^{-1}$)	A_T ($MPa^{-3}s^{-1}$)
0					6.8E-6	2.0E-6
-0.05	1.0E-7	9.0E-7	1.3E-5	12		
-1	5.7E-8	4.2E-7	6.8E-6	8		
-2	3.3E-8	2.4E-7	3.9E-6	10	2.4E-6	1.7E-6
-5	1.2E-8	1.0E-7	1.5E-6	4	1.6E-6	9.3E-7
-10	4.0E-9	**3.2E-8**	5.0E-7	0	4.9E-7	3.5E-7
-20	1.0E-9	**8.0E-9**	1.3E-7	0	1.7E-7	1.2E-7
-30	3.0E-10	**2.3E-9**	3.7E-8	4	5.1E-8	3.7E-8
-40	7.0E-11	**5.5E-10**	8.7E-9	2	1.4E-8	1.0E-8
-50	**1.2E-11**	**9.5E-11**	1.5E-9	1	3.6E-9	2.6E-9

Another common source of parameters for what authors call the Glen flow law is from the third edition of the textbook "Physics of Glaciers" by Paterson (1994). Paterson (1994) adopted the constitutive form from Glen (1955), and rationalised values of n and activation energy, Q, from Weertman (1973), with strain rates informed by laboratory data, most particularly the summary data of Budd & Jacka (1989), and by data from borehole and tunnel closure and from ice-shelf inversions. An increase in the value of Q at temperatures above approximately -10°C is clear in Figure 7 of Budd & Jacka (1989), and a similar, although less extreme pattern is evident in the data presented in Table 5.2 of Paterson (1994) and Table 3.4 of Cuffey & Paterson (2010), who represent the effect of temperature through a table of creep parameters, A_T (note this is just called A in these books), where A_T combines the pre-exponential scaling factor A and the Arrhenius term. This representation takes the form

$$\dot{\varepsilon} = A_T \sigma^n, \tag{S1}$$

where

$$A_T = A\exp\left(-\frac{Q}{\mathrm{R}T}\right). \qquad (S2)$$

For temperatures of 0, -2, and -10°C, Paterson (1994) averaged up to six values from previous laboratory experiments and field measurements to derive the recommended A_{T} values. The A_{T} values for other distinct temperatures were estimated based on an average value at -10°C and an activation energy of 60 kJ/mol (Weertman, 1973). Cuffey & Paterson (2010) updated the table. Values of A_{T} from Paterson (1994), Cuffey and Paterson (2010), and from calculation based on data provided by Budd & Jacka (1989) are shown in Table S1. In Table S1, the first two columns are octahedral minimum strain rates (s^{-1}) from experiments at octahedral shear stresses of 0.2 and 0.4 MPa, adopted from Table 3 of Budd & Jacka (1989). The third column is the average of A_{T} values calculated from the data collected at octahedral shear stresses of 0.2 MPa and 0.4 MPa using Eq. S1 and n = 3, with errors (the ratio between the difference in A_T values calculated at octahedral shear stresses of 0.2 and 0.4 MPa and the averaged A_T value in percentage) shown in the fourth column. The A_{T} values from Paterson (1994) and Cuffey and Paterson (2010) are shown in the fifth and sixth columns from the left. The A_{T} values all correspond to flow laws that relate octahedral shear stresses to octahedral shear strain rates (see Section 1.2).

Paterson (1994) and Cuffey and Paterson (2010) both suggest a change in activation energy for temperatures warmer than -10°C. Paterson (1994) uses an activation energy of 139 kJ/mol (from Weertman, 1973, Table 2) and Cuffey and Paterson (2010) use 115kJ/mol. Neither reference indicates which of the A_{T} values above -10°C should be used as the basis for a flow law for these warm temperatures. Kuiper et al. (2020) list parameters for a Glen flow law that can be used for cold (< 263 K) and warm (> 263 K) temperatures. Paterson (1994) is cited as the source, but we

cannot reproduce Kuiper's A values from Paterson (1994). Table S2 shows parameters for three versions of the Glen flow law with different parameters for cold (< 263 K) and warm (> 263 K) temperatures; these parameters are adopted from Paterson (1994), Cuffey & Paterson (2010), and Kuiper et al (2020). The Glen flow laws for warm temperatures from Paterson (1994) and Cuffey & Paterson (2010) use the A_{T} values from -5°C. In Table S2, the A values are shown for both an octahedral ($A_{\mathrm{oct.}}$) and axial (A_{axial}) flow laws: the values in the source literature are those for the octahedral flow laws (see Section 1.2).

Table S2. Glen flow law parameters from the literature

		Paterson (1994)	Cuffey & Paterson (2010)	Kuiper et al. (2020)
High T (>263 K)	n	3	3	3
	Q (kJ/mol)	139	115	139
	$A_{\mathrm{oct.}}$ ($MPa^{-3}s^{-1}$)	2.05E21	2.50E16	1.73E21
	A_{axial} ($MPa^{-3}s^{-1}$)	3.04E20	3.70E15	2.56E20
Low T (<263 K)	n	3	3	3
	Q	60	60	60
	$A_{\mathrm{oct.}}$ ($MPa^{-3}s^{-1}$)	4.11E5	2.94E5	3.60E5
	A_{axial} ($MPa^{-3}s^{-1}$)	6.09E4	4.36E4	5.33E4

Strain rates as a function of temperature (for a given stress) are plotted in Fig. S1, using the three different sets of flow-law parameters in Table S2 and parameters derived from the data presented in Budd & Jacka (1989), as shown in Table S1. Figure S1 shows the broad similarity of these different Glen flow law parameterisations and resolves an uncertainty as to whether the A or A_{T} values reported in some of the literature relate to octahedral or axial flow laws (see Section 1.2).

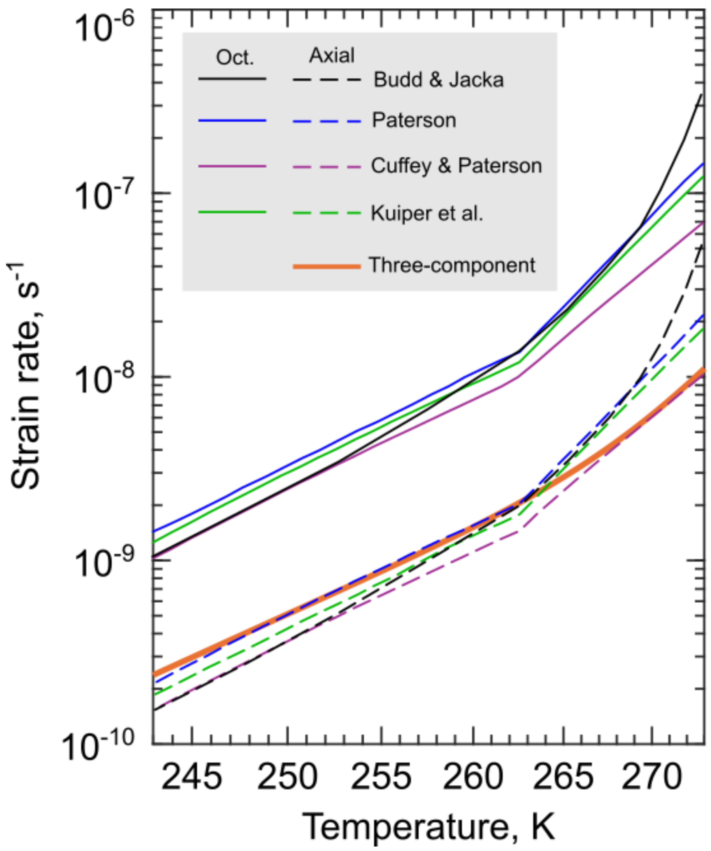

Figure S1. Strain rates for octahedral and axial Glen flow-law relations. The thin solid lines show octahedral strain rates based on the Glen flow law, using parameters from various published sources (Budd & Jacka, 1989; Cuffey & Paterson, 2010; Kuiper et al., 2020; Paterson, 1994) at an octahedral shear stress of 0.3 MPa. The thin dashed lines show axial strain rates at an axial stress of 0.3 MPa. For comparison, a thick solid orange line is included, representing predictions of axial strain rate using the three-component flow law at an axial stress of 0.3 MPa and a grain size of 2 mm (from Fig. 2 in the main text).

1.2 Axial versus octahedral flow laws

Direct measurements from axial compression experiments are normally of differential stress (axial stress minus confining pressure = axial stress for unconfined experiments) and axial strain rate. For simplicity of writing, these are called axial stress and axial strain rate here. Researchers

with a rock mechanics background (e.g., Durham et al., 1983; Goldsby & Kohlstedt, 1997) generally use only the axial measurements. Some glaciological researchers calculate octahedral stress and strain rate (e.g., Jacka & Maccagnan, 1984) from the axial data and this has value for comparison with data from natural ice and also for cross comparison of experiments with differing kinematics (compression, extension, direct shear, torsion). Glen (1955) presents both axial and octahedral data. Flow laws that use axial stress and strain rate versus octahedral stress and strain rate will have different values of scaling constant, A (Budd & Jacka, 1989).

Both stress and strain rate in Table 3 of Budd & Jacka (1989) are clearly labelled as octahedral. Neither Paterson (1994) nor Cuffey & Paterson (2010) state explicitly whether the A values relate to an octahedral flow law, but the reliance on laboratory data from Budd and Jacka (1989) and the nature of the discussion of the relation between flow laws derived by Nye (1953) and Glen (1955) suggests strongly that the A values relate to an octahedral flow law. Kuiper et al (2020) compared the Glen flow law with the Goldsby-Kohlstedt flow law, which is an axial flow law, and make no statement as to whether the values in the Glen flow law are relevant to axial or octahedral stresses and strain rates. Strain rates calculated using the constitutive equation of the Glen flow law with parameter values reported from different sources, including Paterson (1994), Cuffey & Paterson (2010), and Kuiper et al (2020) are similar, and they are close to those calculated using octahedral data reported in Budd & Jacka (1989) (Fig. S1). This observation suggests that the published parameters are all for octahedral flow laws. The conversion between an axial and octahedral flow law is straight forward. If a flow law uses axial stress, σ_{axial}, and axial strain rate, $\dot{\varepsilon}_{\mathrm{axial}}$, then, the conversion to a flow law using octahedral shear stress, $\tau_{\mathrm{oct.}}$, and octahedral shear strain rate, $\dot{\varepsilon}_{\mathrm{oct.}}$, can be calculated using

$$\dot{\varepsilon}_{\text{axial}} = A_{\text{axial}}\sigma_{\text{axial}}^{n} \exp\left(-\frac{Q}{RT}\right), \tag{S3}$$

$$\dot{\varepsilon}_{\text{axial}} = \sqrt{2}\dot{\varepsilon}_{\text{oct.}}, \tag{S4}$$

and

$$\sigma_{\text{axial}} = \frac{3}{\sqrt{2}}\tau_{\text{oct.}} \,. \tag{S5}$$

From equations (1), (2), and (3), we derive the octahedral form of the axial flow law:

$$\dot{\varepsilon}_{\text{oct.}} = \frac{1}{\sqrt{2}}\left(\frac{3}{\sqrt{2}}\right)^{n} A_{\text{axial}}\tau_{\text{oct.}}^{n} \exp\left(-\frac{Q}{RT}\right) \,. \tag{S6}$$

Only the A value needs to be changed through the conversion

$$A_{\text{oct}} = \frac{1}{\sqrt{2}}\left(\frac{3}{\sqrt{2}}\right)^{n} A_{\text{axial}}, \tag{S7}$$

and likewise

$$A_{\text{axial}} = \sqrt{2}\left(\frac{\sqrt{2}}{3}\right)^{n} A_{\text{oct.}} \tag{S8}$$

Therefore, if n = 3, then the octahedral value of A is 6.75 times the axial value.

Some studies (e.g., McCormack et al., 2022; Treverrow et al., 2015) use effective shear stress and strain rate (Budd et al., 2013) as an alternative to octahedral shear stress and strain rate. Equations relating effective and octahedral values are presented as Equation 8 in Budd et al (2013). For completeness we include the expression that relates the $A_{\text{eff.}}$ value, for a flow law using effective stress and strain rate, to $A_{\text{oct.}}$

$$A_{\text{eff.}} = \left(\sqrt{\frac{2}{3}}\right)^{n-1} A_{\text{oct.}} \tag{S9}$$

In the main text, we use parameters from Kuiper et al (2020) for the Glen flow law, adjusting these using Eq. S8 to have a value of A for axial data (Table S2) for correct comparison to our axial flow laws. We choose Kuiper et al (2020) as it approximates a "median" Glen flow law (of those in Table S2) and has no change of strain rate at the transition temperature between cold and warm flow laws. Predictions using parameters from Paterson (1994) and Cuffey & Paterson (2010) (Table S2) have small changes in strain rate at the transition temperature. The results of the paper would not be substantially different if we were to use any of the other parameterisations of the Glen flow law discussed here.

2. Mechanical details of additional experiments

Here, we present four additional deformation experiments (PIL264, PIL263, PIL261, PIL252) on ice conducted under uniaxial compression with a confining pressure of ~10 MPa and temperature of -30°C. The average grain size of the starting material is ~300 µm (Fan et al., 2020). These experiments were conducted at the Ice Physics Lab at the University of Pennsylvania.

Experimental runs of PIL264, PIL263, and PIL261 were at strain rates of ~$2.5 \times 10^{-5}\ \mathrm{s}^{-1}$ and PIL252 was at a strain rate of ~$5 \times 10^{-5}\ \mathrm{s}^{-1}$. Mechanical data are presented as stress-strain curves (Fig. S2).

During each experimental run, time, t, and vertical shortening of the ice sample, $s(t)$, were recorded every 3–5 seconds. The true axial strain, $\varepsilon(t)$, was calculated from the initial sample length, L_0, and vertical shortening, $s(t)$, as

$$\varepsilon(t) = -\ln\left(\frac{L_0 - s(t)}{L_0}\right). \tag{S10}$$

The bulk true axial strain rate, $\dot{\varepsilon}(t)$, was calculated from the true axial strain increment during time interval, Δt, as

$$\dot{\varepsilon}(t) = \frac{\varepsilon(t) - \varepsilon(t - \Delta t)}{\Delta t}. \quad \text{(S11)}$$

The bulk axial differential stress, σ, was calculated from the axial load, $F(t)$ (recorded every 3–5 seconds), and cross-sectional area of the ice sample. σ was corrected for the change in cross-sectional area of the sample, assuming constant volume, as

$$\sigma(t) = \frac{F(t)}{\pi R(t)^2}, \quad \text{(S12)}$$

where $R(t)$ is the cross-sectional radius of the sample at time t; $R(t) = R_0\sqrt{\frac{L_0}{L_0 - s(t)}}$.

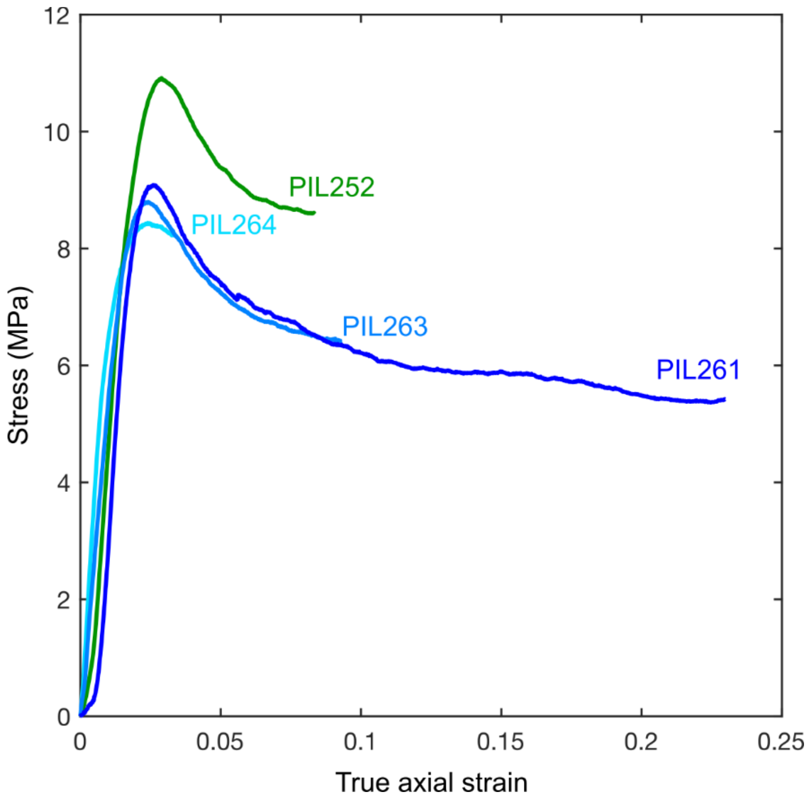

Figure S2. Stress-strain curves for the additional experiments.

3. Previous flow laws give discontinuous predictions at arbitrary threshold temperatures

Figure S3 presents the modelled axial strain rate as a function of stress, temperature, and/or grain size using the Glen flow law (Paterson, 1994) and Goldsby-Kohlstedt flow law (Goldsby & Kohlstedt, 2001) coupled with calibrated parameters (Kuiper et al., 2020) (Table 1). Axial strain rate predicted from Glen flow law is converted from octahedral shear strain rate (Supplementary Information Section 1, Section Mathematical forms of flow laws in the main text). Blue arrows indicate the imposed threshold temperature.

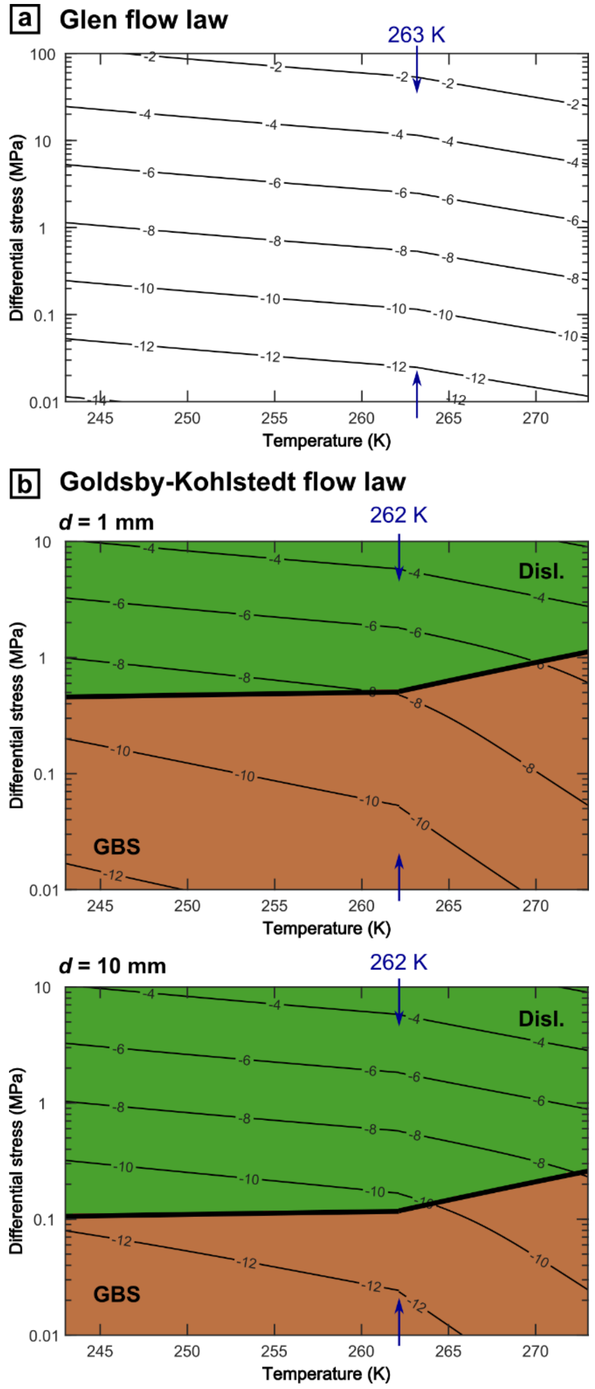

Figure S3. Illustrating predictions of previous flow laws. a and b delineate the interplay among three macroscopic variables (i.e., stress, temperature, and the decimal logarithm of axial strain rate (depicted as contours with thin black lines)), for the Glen flow law and Goldsby-Kohlstedt flow law, respectively.

4. Contribution of different components for the four-component flow law

The four-component flow law contains two grain-size insensitive (GSI) and two grain-size sensitive (GSS) components summed as:

$$\dot{\varepsilon}_{\mathrm{total}} = \dot{\varepsilon}_{\mathrm{GSI1}} + \dot{\varepsilon}_{\mathrm{GSI2}} + \dot{\varepsilon}_{\mathrm{GSS1}} + \dot{\varepsilon}_{\mathrm{GSS2}}. \qquad \text{(S13)}$$

The GSI components have the form of Eq. (2), the GSS components have the form of Eq. (3). We use the Bayesian inference method (Methods) to derive the best-fit parameters of stress exponent, n, grain-size exponent, p, activation energy, Q, and scaling constant, A, for each of the components (Fig. S4). We calculated the ratio between each of the components and the total strain rate across a stress range of 0–5 MPa, temperature range of 243–273 MPa, and grain sizes of 0.1, 1, and 10 mm (Figs. S5–S7). One of the GSI components contribute less than 10^{-19} of the total strain rate for all the conditions (the first row, Figs. S4–S6).

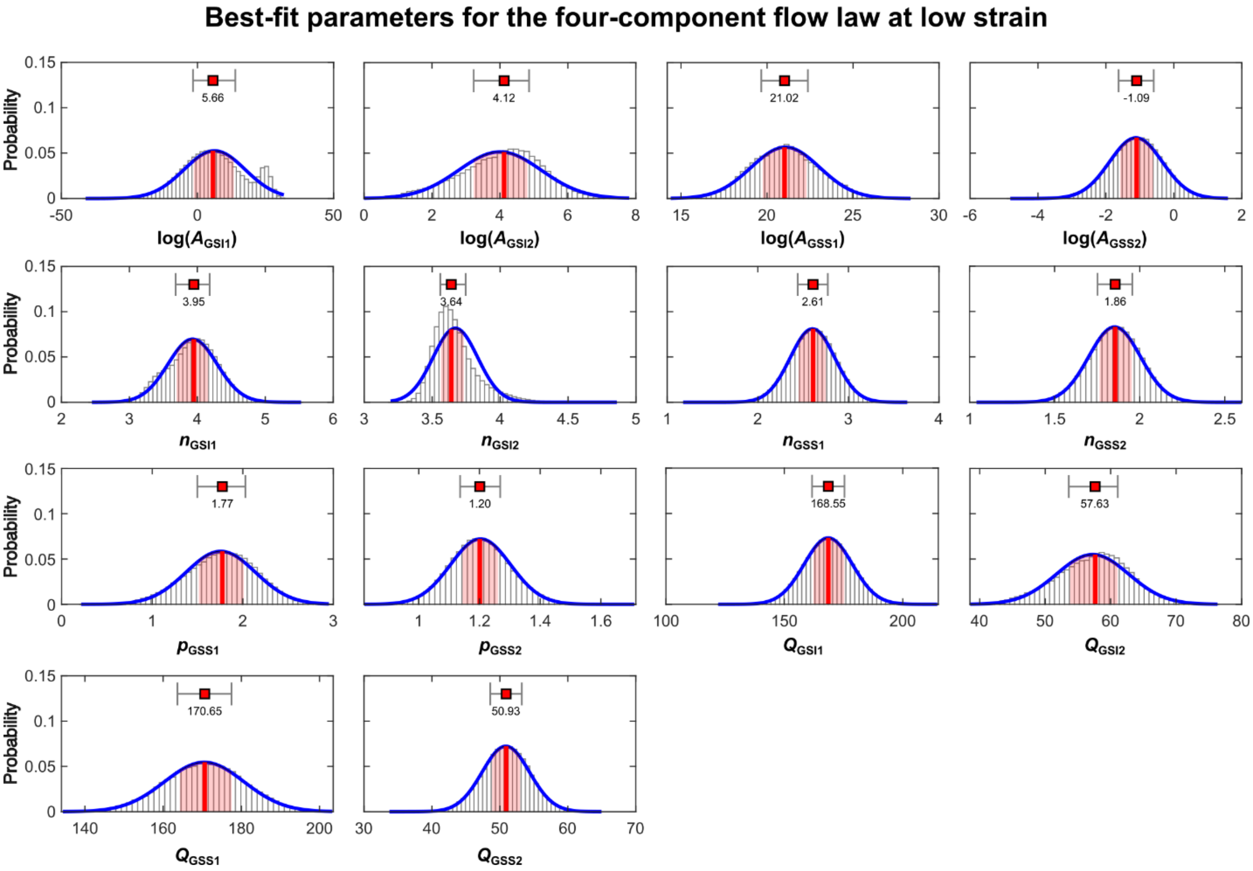

Figure S4. Posterior distributions of parameters for the four-component flow law at low strain, corresponding to the secondary minimum strain rate (for constant load experiments) or peak stress (for constant displacement rate experiments). The posterior distribution is depicted as a histogram for each parameter, superimposed with a fitting normal distribution (blue solid curve). The distribution of each parameter is derived from a dataset comprising 598500 samples generated from Bayesian modelling. The interquartile range is emphasised with a light-red shaded area on the histogram and corresponds to the horizontal error bar above. The median is denoted by a pronounced vertical line on the histogram and a red square on the error bar. Quartile values are indicated in grey, with the median highlighted in red.

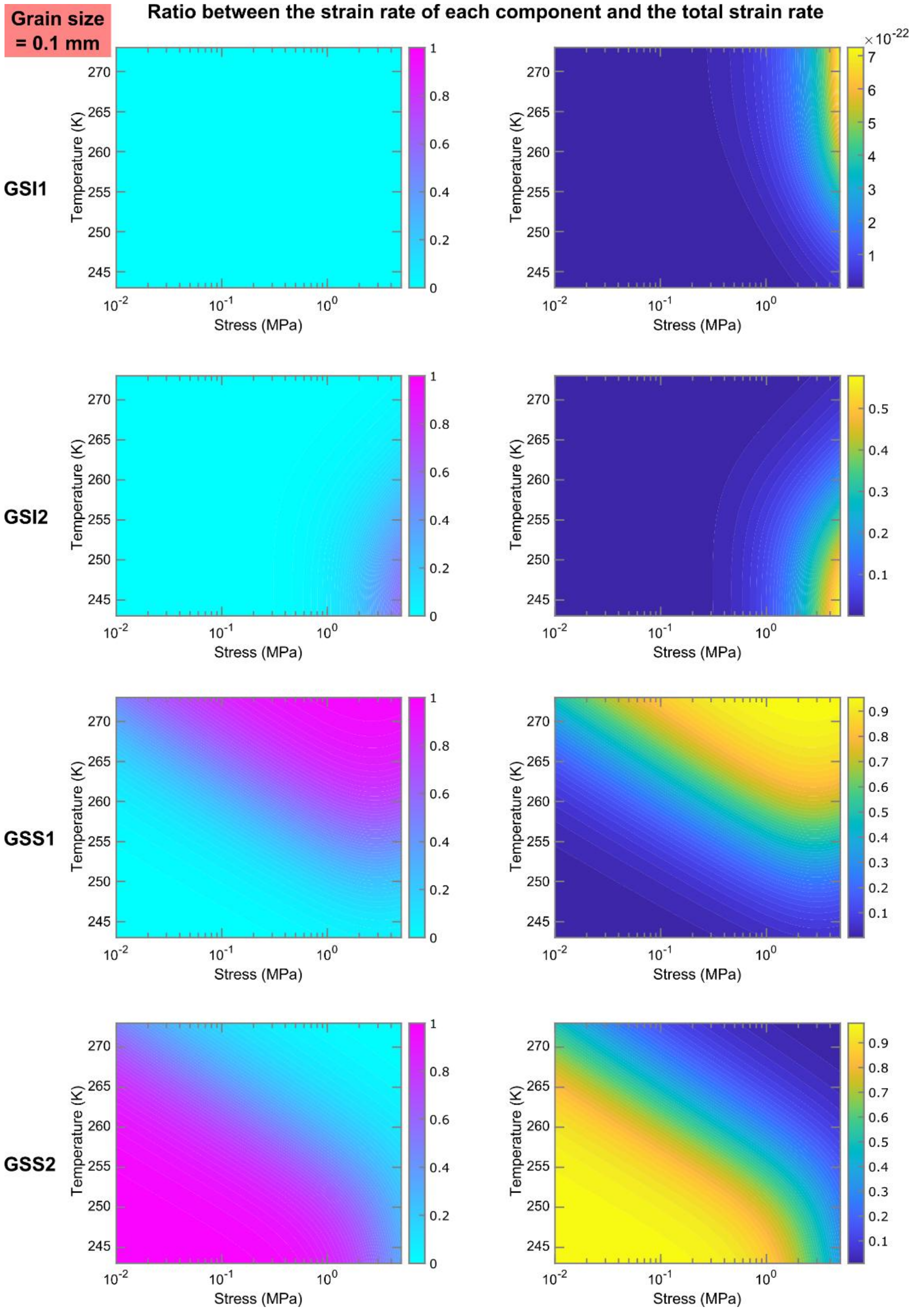

Figure S5. Contributions of each component to the total strain rate for the four-component flow law at a grain size of 100 μm as functions of temperature and stress. Each row corresponds to a different component. The left and right columns display the same data but use differently scaled colour bars. The left column uses a uniformly scaled colour bar across all components, while the right column has individually scaled colour bars to highlight more details.

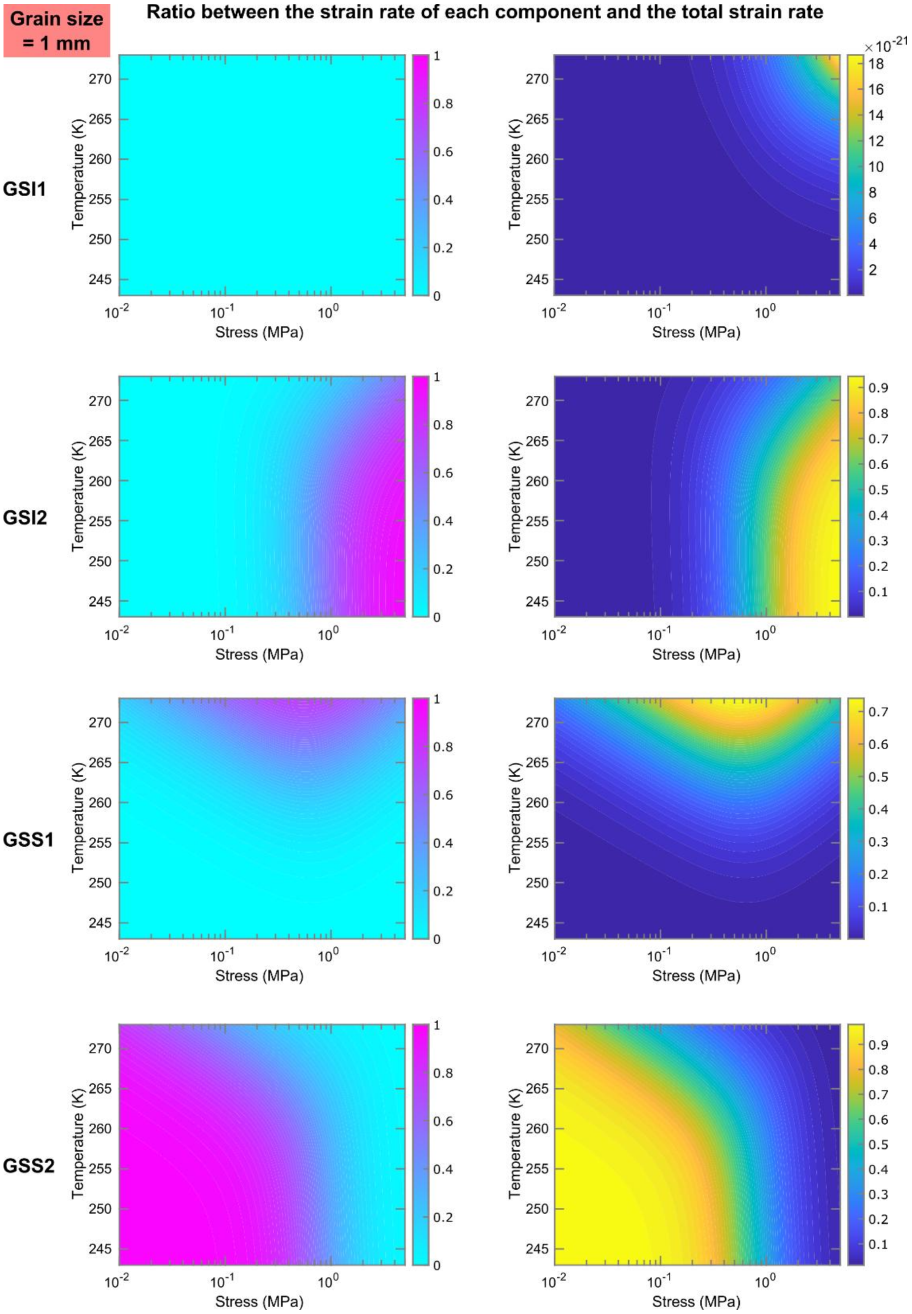

Figure S6. Contribution of each component to the total strain rate for the four-component flow law at a grain size of 1 mm. Descriptions of the plots are the same as in Fig. S5.

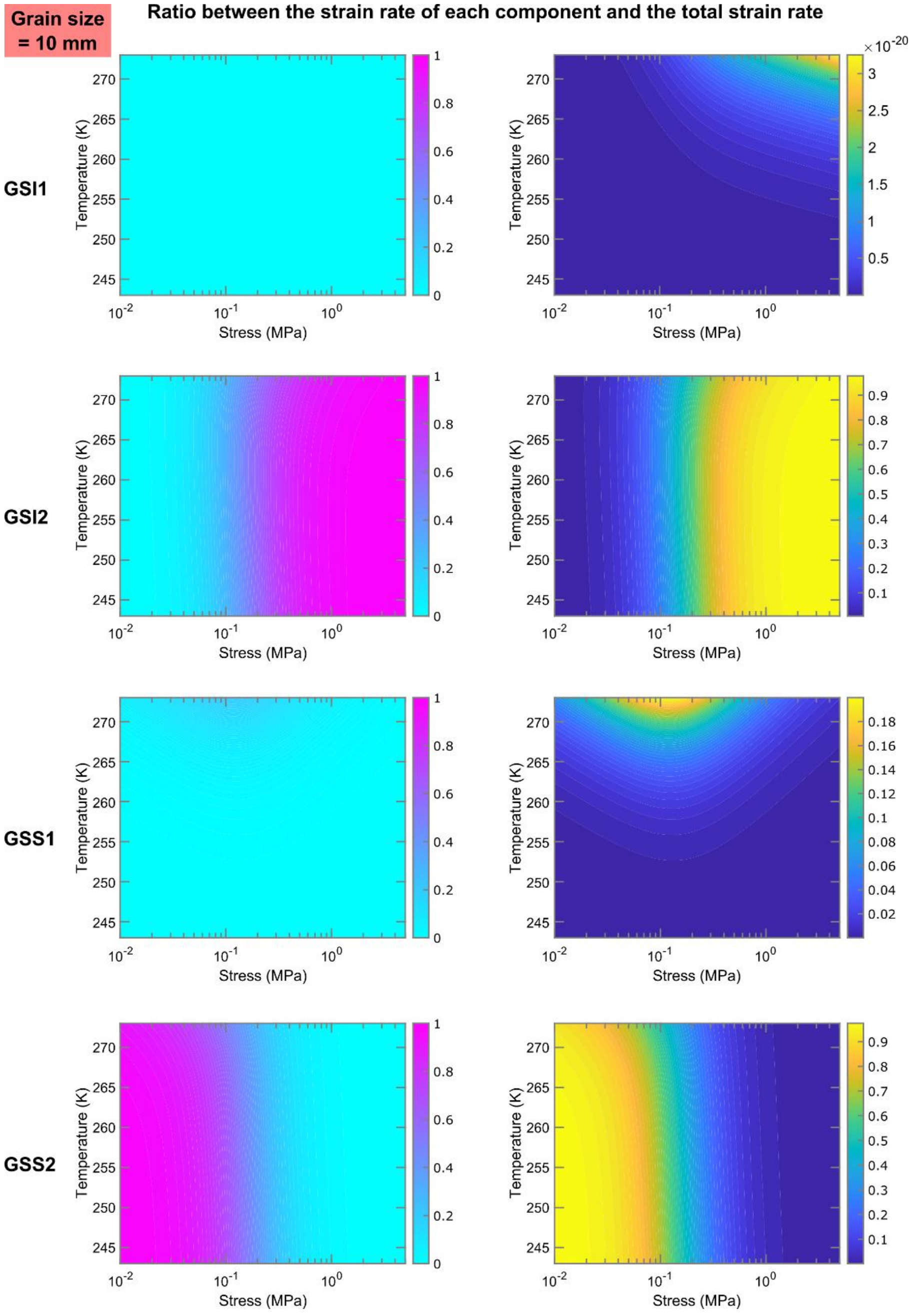

Figure S7. Contribution of each component to the total strain rate for the four-component flow law at a grain size of 10 mm. Descriptions of the plots are the same as in Fig. S5.

5. Comparison of posterior distributions of parameters for three-component flow laws

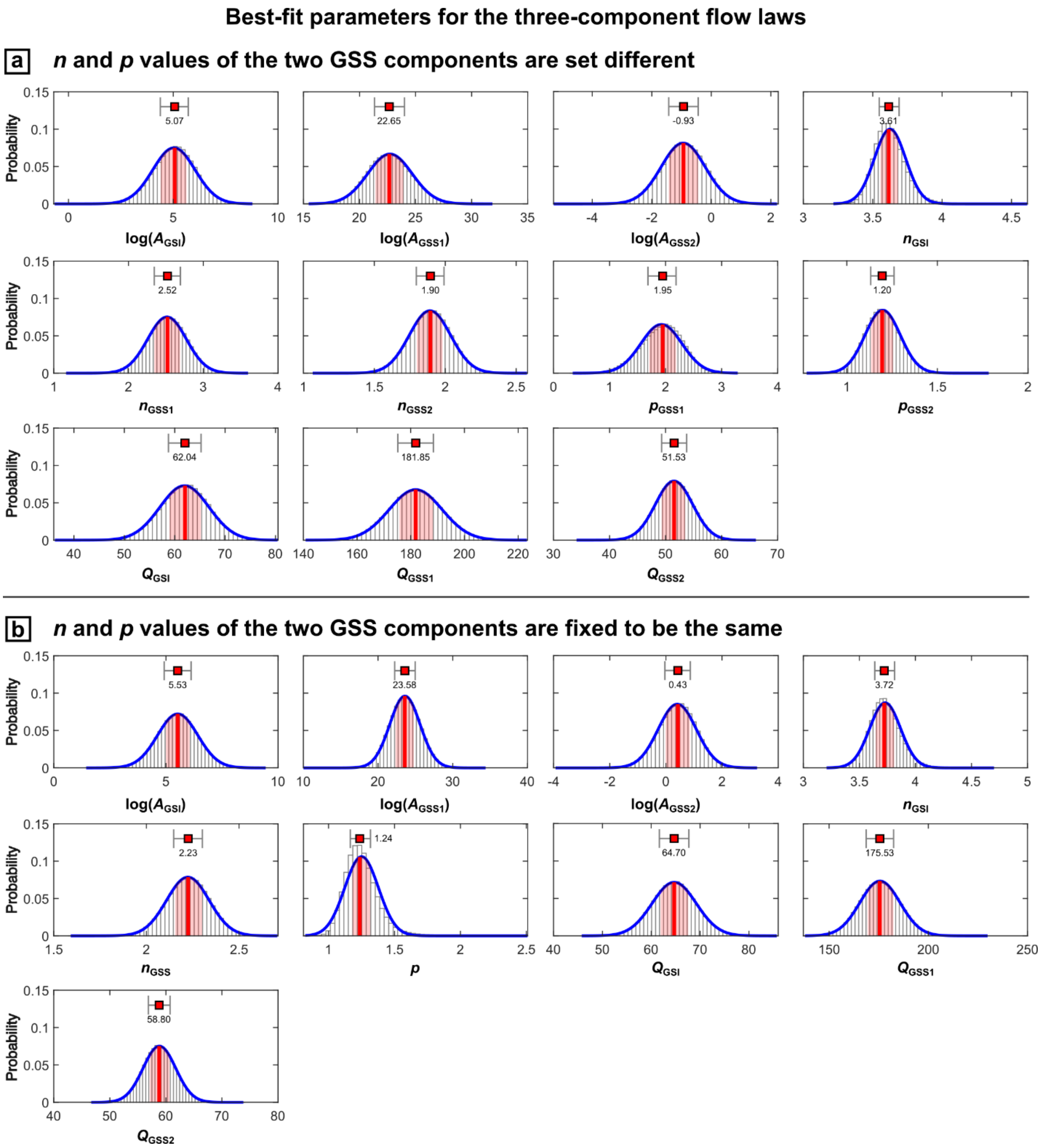

Figure S8. Posterior distributions of parameters for the three-component flow laws at low strain, with n and p set to be **a**, different, and **b**, the same across the two GSS components. The posterior distribution is depicted as a histogram for each parameter, superimposed with a fitting

normal distribution (blue solid curve). The distribution of each parameter in **a** and **b** is derived from a dataset comprising 598500 samples generated from Bayesian modelling. The interquartile range is emphasised with a light-red shaded area on the histogram and corresponds to the horizontal error bar above. The median is denoted by a pronounced vertical line on the histogram and a red square on the error bar. Quartile values are indicated in grey, with the median highlighted in red.

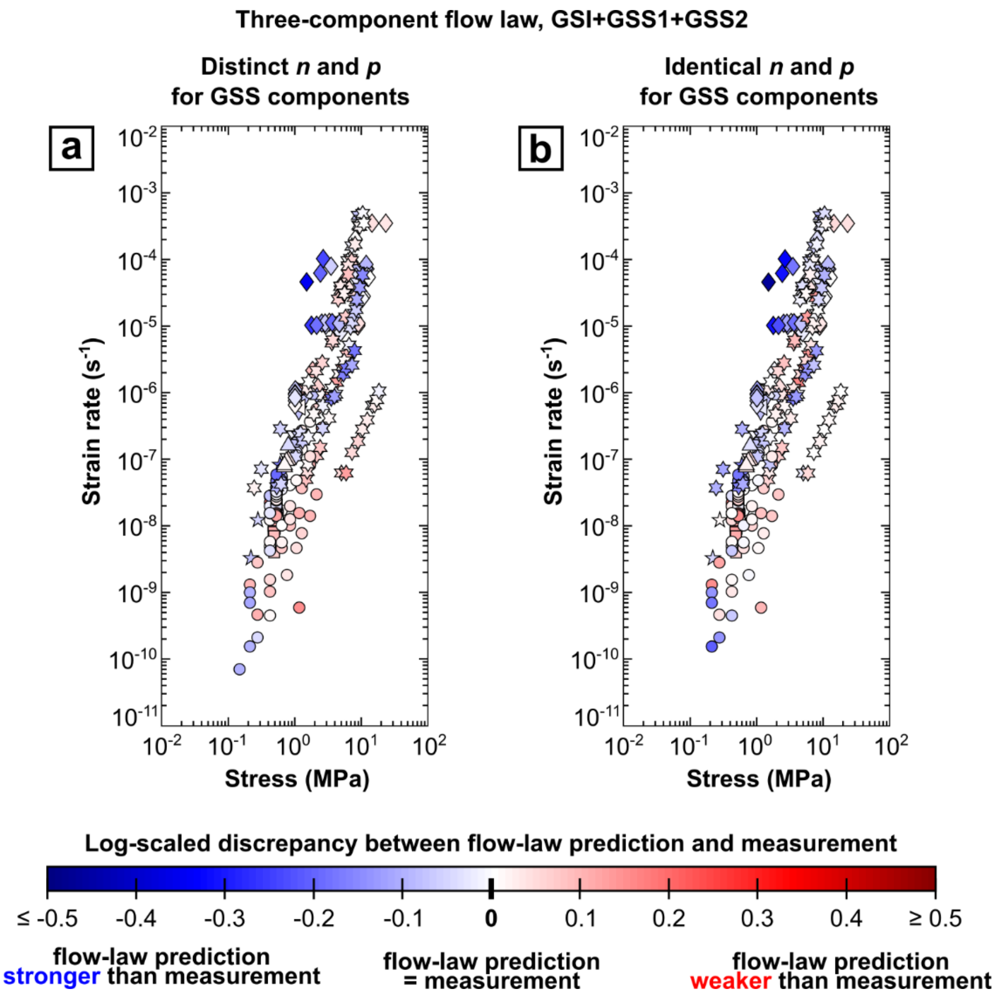

Figure S9. Comparison of predictions from three-component flow laws at low strain, using both distinct and identical n and p values for the GSS components, against experimental measurements.

6. MCMC convergence diagnostics

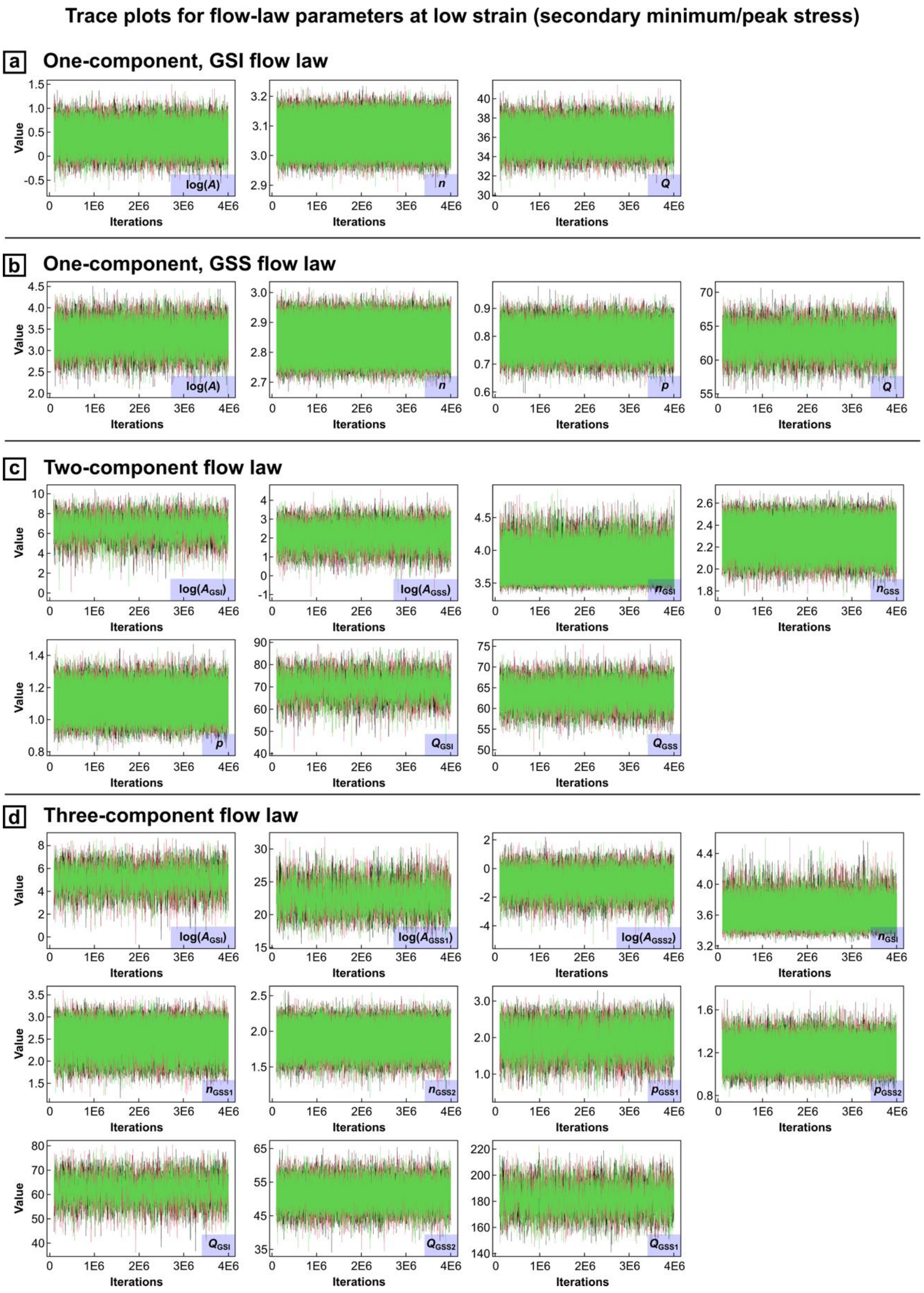

Figure S10. Trace plots of MCMC chains for parameters of flow laws at low strain, corresponding to the secondary minimum (for constant-load experiments) or peak stress (for constant displacement rate experiments). Each trace plot represents the sampled values of a parameter over the iterations of the MCMC process, allowing for the assessment of chain convergence and mixing. Each trace plot contains lines with three different colours (red, green, and black); each line corresponds to one of the three MCMC chains.

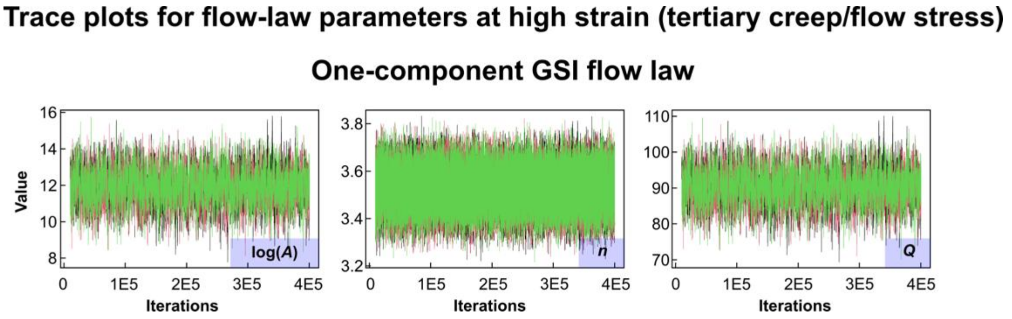

Figure S11. Trace plots of MCMC chains for parameters of best-fit one-component GSI flow law at high strain, corresponding to tertiary creep (for constant load experiments) or flow stress (for constant displacement rate experiments). Descriptions of the plots are the same as in Fig. S10.

7. Bayesian-inference results with inputs containing data from high-stress (>1.5 MPa) experiments that use an unconfined medium

We utilised data from 411 experiments (Supplementary Table 4) to constrain ice-flow mechanics at low strain, particularly relevant to peak stress or the secondary-creep (minimum) strain rate. We then used data from an additional 162 experiments (Supplementary Table 5) to constrain ice-flow mechanics at high strain, steady-state conditions, which correspond to the flow stress or tertiary-creep strain rate. Bayesian inference predicts a normal distribution of the posteriors for

n, Q, p and log (A); prior and posterior statistics of n, Q, A, and p for each flow-law component are shown in Table S3.

Table S3. Details of flow law parameters when high-stress, unconfined experiments are considered

Deformation stage	Flow law	#Parameter	*Prior distribution	*Prior parameter				†Posterior statistics		
				μ	s^2	min	max	Lower quartile	**Median**	Higher quartile
Low strain (1-2%), peak stress or secondary-minimum strain rate	One-component flow law, GSI	n	$TN(\mu, s^2, min, max)$	4	100	0	10	3.3	**3.4**	3.4
		Q	$TN(\mu, s^2, min, max)$	60	100	0	250	41	**42**	43
		log (A)	$U(min, max)$	N/A	N/A	-50	50	1.47	**1.61**	1.75
	One-component flow law, GSS	n	$TN(\mu, s^2, min, max)$	4	100	0	10	3.2	**3.3**	3.3
		Q	$TN(\mu, s^2, min, max)$	60	100	0	250	62	**63**	64
		p	$TN(\mu, s^2, min, max)$	1.4	100	0	10	0.6	**0.6**	0.6
		log (A)	$U(min, max)$	N/A	N/A	-50	50	3.90	**4.09**	4.27
	Two-component flow law, GSI+GSS	n_{GSI}	$TN(\mu, s^2, min, max)$	4	0.1	0	10	4.0	**4.1**	4.2
		n_{GSS}	$TN(\mu, s^2, min, max)$	1.8	100	0	10	2.0	**2.1**	2.1
		p	$TN(\mu, s^2, min, max)$	1.4	100	0	5	1.1	**1.1**	1.2
		Q_{GSI}	$TN(\mu, s^2, min, max)$	60	100	0	250	85	**88**	90
		Q_{GSS}	$TN(\mu, s^2, min, max)$	60	100	0	250	57	**59**	61
		log (A_{GSI})	$U(min, max)$	N/A	N/A	-50	50	9.73	**10.20**	10.69
		log (A_{GSS})	$U(min, max)$	N/A	N/A	-50	50	0.74	**1.11**	1.47
	Three-component flow law, GSI+GSS1+GSS2	n_{GSI}	$TN(\mu, s^2, min, max)$	4	0.1	0	10	4.0	**4.1**	4.2
		n_{GSS1}	$TN(\mu, s^2, min, max)$	1.8	100	0	10	1.8	**2.0**	2.2
		p_{GSS1}	$TN(\mu, s^2, min, max)$	1.4	100	0	5	2.1	**2.3**	2.5
		n_{GSS2}	$TN(\mu, s^2, min, max)$	1.8	100	0	10	1.8	**1.9**	2.0
		p_{GSS2}	$TN(\mu, s^2, min, max)$	1.4	100	0	5	1.1	**1.1**	1.2
		Q_{GSI}	$TN(\mu, s^2, min, max)$	60	100	0	250	81	**84**	86
		Q_{GSS1}	$TN(\mu, s^2, min, max)$	180	100	0	250	178	**184**	191
		Q_{GSS2}	$TN(\mu, s^2, min, max)$	50	100	0	250	48	**50**	52
		log (A_{GSI})	$U(min, max)$	N/A	N/A	-50	50	8.86	**9.38**	9.89

		log (A_{GSS1})	$U(min, max)$	N/A	N/A	-50	50	20.63	**21.89**	23.13
		log (A_{GSS2})	$U(min, max)$	N/A	N/A	-50	50	-1.25	**-0.80**	-0.36
	Three-component flow law with the same n and p values for GSS components	n_{GSI}	$TN(\mu, s^2, min, max)$	4	0.1	0	10	4.1	**4.2**	4.3
		n_{GSS}	$TN(\mu, s^2, min, max)$	1.8	100	0	10	2.0	**2.0**	2.1
		p	$TN(\mu, s^2, min, max)$	1.4	100	0	5	1.1	**1.2**	1.2
		Q_{GSI}	$TN(\mu, s^2, min, max)$	60	100	0	250	82	**85**	87
		Q_{GSS1}	$TN(\mu, s^2, min, max)$	180	100	0	250	171	**178**	185
		Q_{GSS2}	$TN(\mu, s^2, min, max)$	50	100	0	250	53	**54**	56
		log (A_{GSI})	$U(min, max)$	N/A	N/A	-50	50	9.00	**9.48**	9.99
		log (A_{GSS1})	$U(min, max)$	N/A	N/A	-50	50	22.83	**24.18**	25.55
		log (A_{GSS2})	$U(min, max)$	N/A	N/A	-50	50	-0.57	**0.16**	0.23
High strain (≥ ~8%), flow stress or tertiary-creep strain rate	One-component flow law, GSI	n	$TN(\mu, s^2, min, max)$	4	100	0	10	3.5	**3.5**	3.6
		Q	$TN(\mu, s^2, min, max)$	60	100	0	250	87	**90**	93
		log (A)	$U(min, max)$	N/A	N/A	0	50	11.28	**11.91**	12.54

# The scaling constant, A, has a unit of $\mathrm{MPa}^{-n}\mathrm{m}^{p}\mathrm{s}^{-1}$; the activation energy, Q, has a unit of kJmol^{-1}. The logarithms are calculated to base 10. The flow law parameters are used for predicting axial strain rate with an input of axial stress, temperature, and grain size.

* $TN(\mu, s^2, min, max)$ represents a normal distribution truncated between specified minimum and maximum bounds, with mean, μ, and variance, s^2. $U(min, max)$ represents a uniform distribution where all values between the specified minimum and maximum bounds are equally likely.

† The median, lower quartile, and upper quartile values of each parameter are derived from the posterior distributions obtained through Bayesian statistical modelling.

References

Budd, W.F., & Jacka, T. H. (1989). A review of ice rheology for ice sheet modelling. *Cold Regions Science and Technology*, *16*(2), 107–144. https://doi.org/10.1016/0165-232X(89)90014-1

Budd, William F., Warner, R. C., Jacka, T. H., Li, J., & Treverrow, A. (2013). Ice flow relations for stress and strain-rate components from combined shear and compression laboratory experiments. *Journal of Glaciology*, *59*(214), 374–392. https://doi.org/10.3189/2013JoG12J106

Cuffey, K. M., & Paterson, W. S. B. (2010). *The Physics of Glaciers* (4th ed.). Butterworth-Heinemann.

Durham, W. B., Heard, H. C., & Kirby, S. H. (1983). Experimental deformation of polycrystalline H2O ice at high pressure and low temperature: Preliminary results. *Journal of Geophysical Research*, *88*(S01), B377–B392. https://doi.org/10.1029/JB088iS01p0B377

Fan, S., Hager, T. F., Prior, D. J., Cross, A. J., Goldsby, D. L., Qi, C., et al. (2020). Temperature and strain controls on ice deformation mechanisms: insights from the microstructures of samples deformed to progressively higher strains at −10, −20 and −30 °C. *The Cryosphere*, *14*(11), 3875–3905. https://doi.org/10.5194/tc-14-3875-2020

Glen, J. W. (1952). Experiments on the Deformation of Ice. *Journal of Glaciology*, *2*(12), 111–114. https://doi.org/10.1017/s0022143000034067

Glen, J. W. (1953). Rate of Flow of Polycrystalline Ice. *Nature*, *172*(4381), 721–722. https://doi.org/10.1038/172721a0

Glen, J. W. (1955). The creep of polycrystalline ice. *Proceedings of the Royal Society of London. Series A. Mathematical and Physical Sciences*, *228*(1175), 519–538. https://doi.org/10.1098/rspa.1955.0066

Goldsby, D. L., & Kohlstedt, D. L. (1997). Grain boundary sliding in fine-grained Ice I. *Scripta Materialia*, *37*(9), 1399–1406. https://doi.org/10.1016/S1359-6462(97)00246-7

Goldsby, D. L., & Kohlstedt, D. L. (2001). Superplastic deformation of ice: Experimental observations. *Journal of Geophysical Research: Solid Earth*, *106*(B6), 11017–11030. https://doi.org/10.1029/2000jb900336

Jacka, T. H., & Maccagnan, M. (1984). Ice crystallographic and strain rate changes with strain in compression and extension. *Cold Regions Science and Technology*, *8*(3), 269–286. https://doi.org/10.1016/0165-232X(84)90058-2

Kuiper, E. J. N., De Bresser, J. H. P., Drury, M. R., Eichler, J., Pennock, G. M., & Weikusat, I. (2020). Using a composite flow law to model deformation in the NEEM deep ice core, Greenland-Part 2: The role of grain size and premelting on ice deformation at high homologous temperature. *Cryosphere*, *14*(7), 2449–2467. https://doi.org/10.5194/tc-14-2449-2020

McCormack, F. S., Warner, R. C., Seroussi, H., Dow, C. F., Roberts, J. L., & Treverrow, A. (2022). Modeling the Deformation Regime of Thwaites Glacier, West Antarctica, Using a Simple Flow Relation for Ice Anisotropy (ESTAR). *Journal of Geophysical Research: Earth Surface*, *127*(3). https://doi.org/10.1029/2021JF006332

Nye, J. F. (1953). The flow law of ice from measurements in glacier tunnels, laboratory experiments and the Jungfraufirn borehole experiment. *Proceedings of the Royal Society of*

London. Series A. Mathematical and Physical Sciences, *219*(1139), 477–489. https://doi.org/10.1098/rspa.1953.0161

Paterson, W. S. B. (1994). *The Physics of Glaciers* (third). Elsevier.

Treverrow, A., Warner, R. C., Budd, W. F., Jacka, T. H., & Roberts, J. L. (2015). Modelled stress distributions at the Dome Summit South borehole, Law Dome, East Antarctica: A comparison of anisotropic ice flow relations. *Journal of Glaciology*, *61*(229), 987–1004. https://doi.org/10.3189/2015JoG14J198

Weertman, J. (1973). Creep of ice. In E. Whalley, S. J. Jones, & L. W. Gold (Eds.), *Physics and chemistry of ice* (pp. 320–337). Ottawa,Canada: Royal Society of Canada.
