# Supplementary figures and images for: Flow laws for ice constrained by 70 years of laboratory experiments
Source: Nat Geosci. 2025 Mar 28;18(4):296–304. doi: 10.1038/s41561-025-01661-z (PMC11981940; doi:10.1038/s41561-025-01661-z)

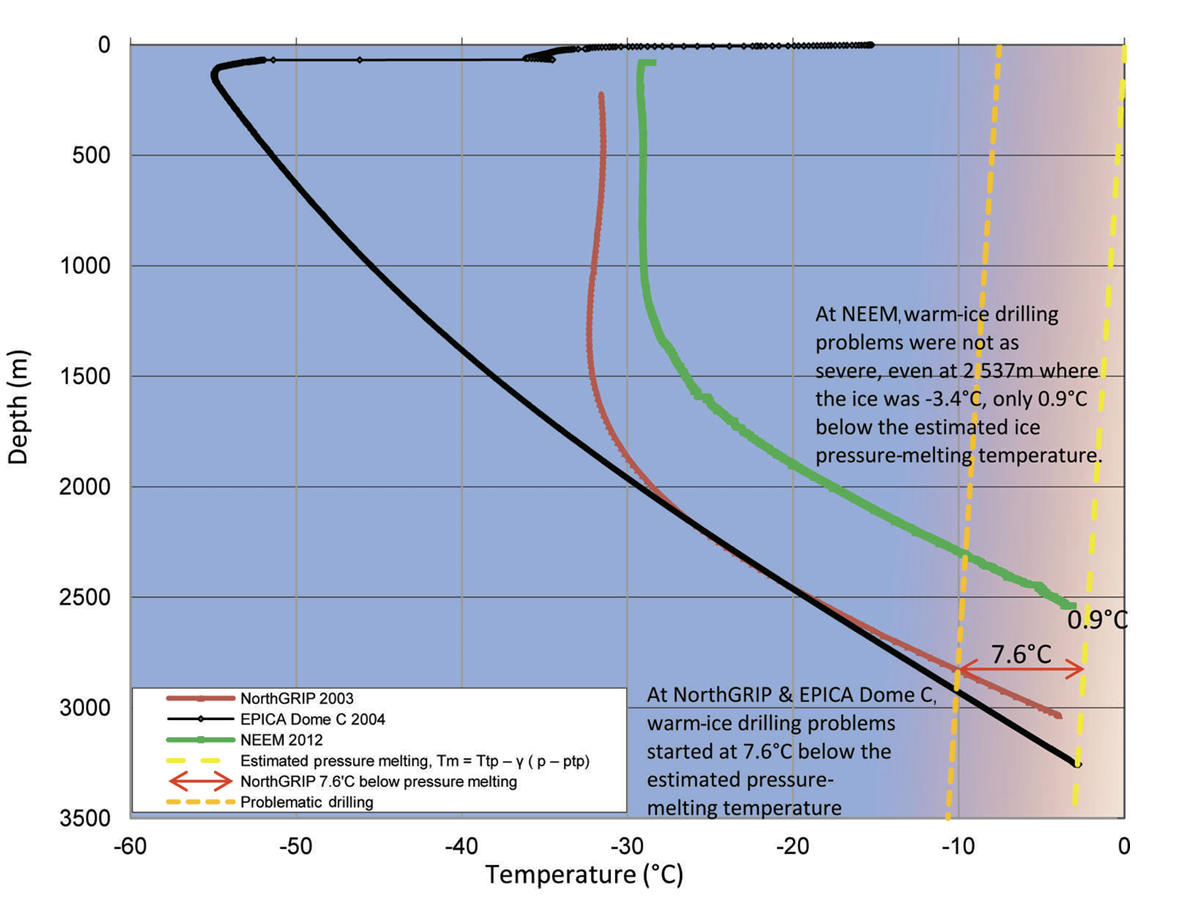

Supplement: Supplementary file 4 — Digitized data for published experiments and temperature measurements as a function of depth for the NEEM ice core. [file 41561_2025_1661_MOESM4_ESM.zip › Supplement_data2/NEEM_temperature/NEEM_temp.png]

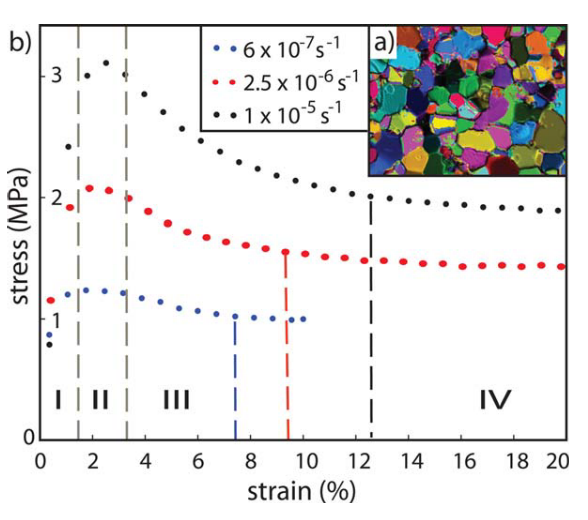

Supplement: Supplementary file 4 — Digitized data for published experiments and temperature measurements as a function of depth for the NEEM ice core. [file 41561_2025_1661_MOESM4_ESM.zip › Supplement_data2/Mechanical_data/Piazolo_2013/P_2013.PNG]

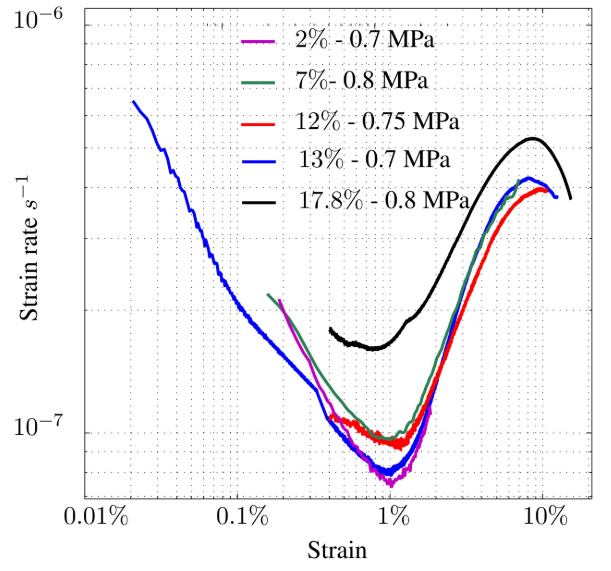

Supplement: Supplementary file 4 — Digitized data for published experiments and temperature measurements as a function of depth for the NEEM ice core. [file 41561_2025_1661_MOESM4_ESM.zip › Supplement_data2/Mechanical_data/Montagnat_2015/M_2015.png]

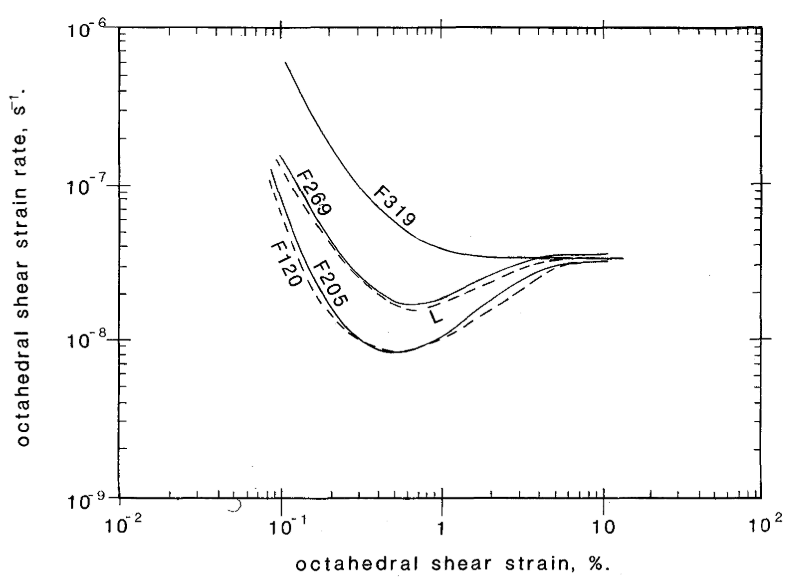

Supplement: Supplementary file 4 — Digitized data for published experiments and temperature measurements as a function of depth for the NEEM ice core. [file 41561_2025_1661_MOESM4_ESM.zip › Supplement_data2/Mechanical_data/Gao_Jacka_1987/G_J_1987.png]

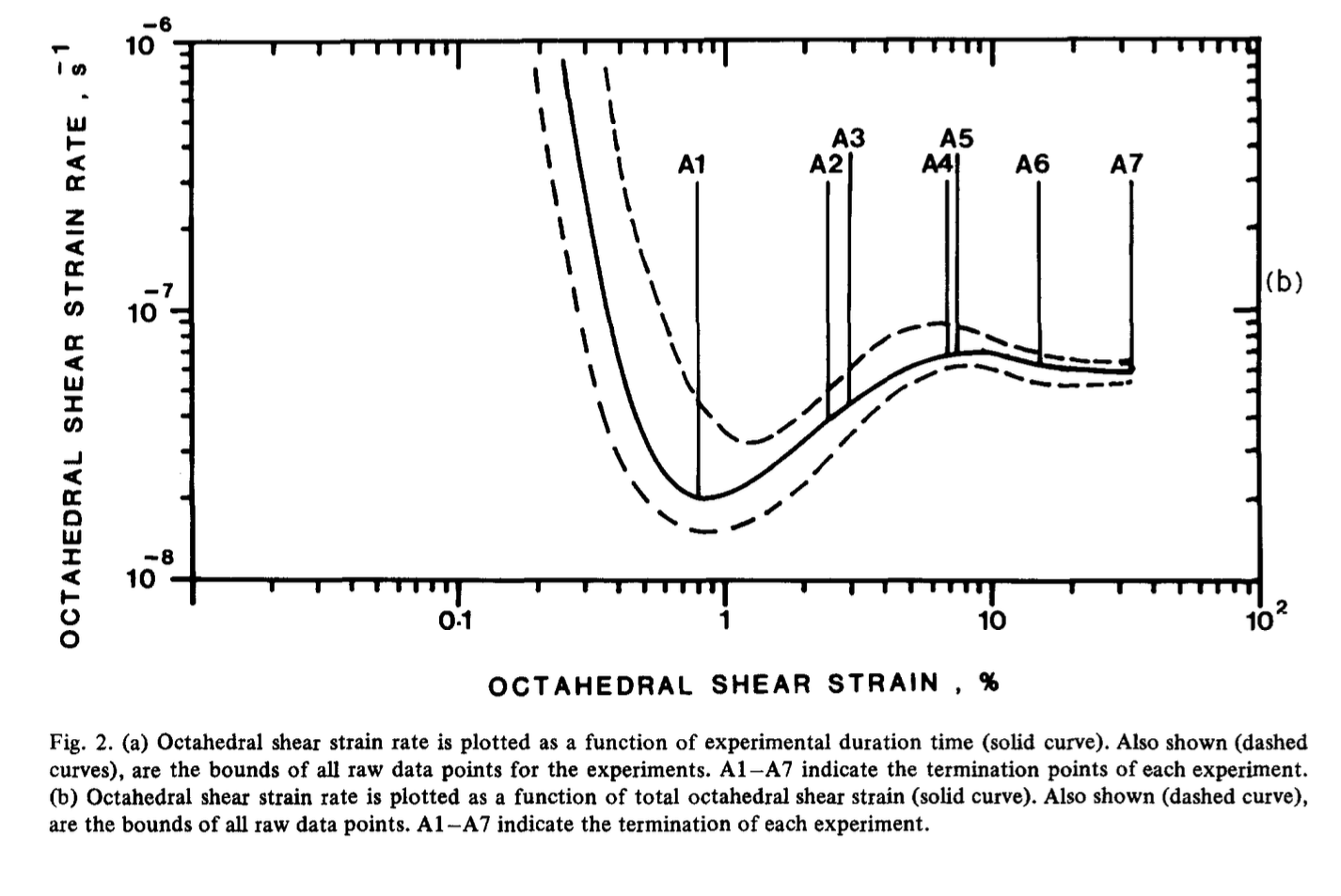

Supplement: Supplementary file 4 — Digitized data for published experiments and temperature measurements as a function of depth for the NEEM ice core. [file 41561_2025_1661_MOESM4_ESM.zip › Supplement_data2/Mechanical_data/Jacka_Maccagnan_1984/Fig. 2.png]

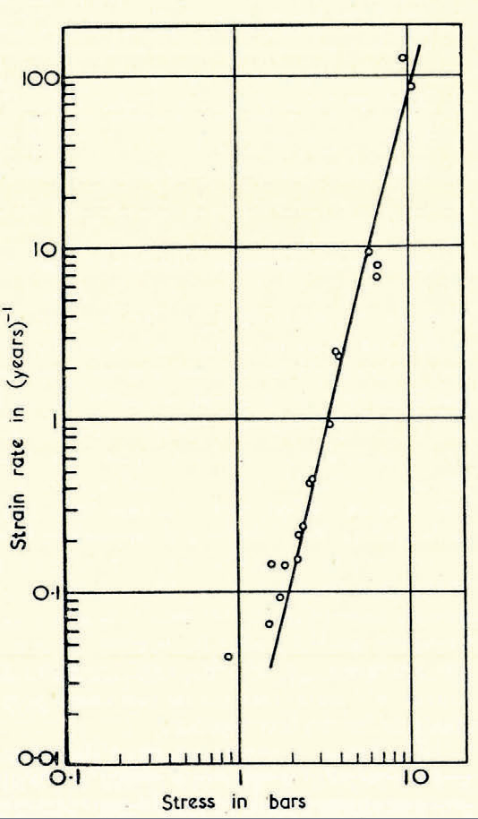

Supplement: Supplementary file 4 — Digitized data for published experiments and temperature measurements as a function of depth for the NEEM ice core. [file 41561_2025_1661_MOESM4_ESM.zip › Supplement_data2/Mechanical_data/Glen_1952/Capture.PNG]

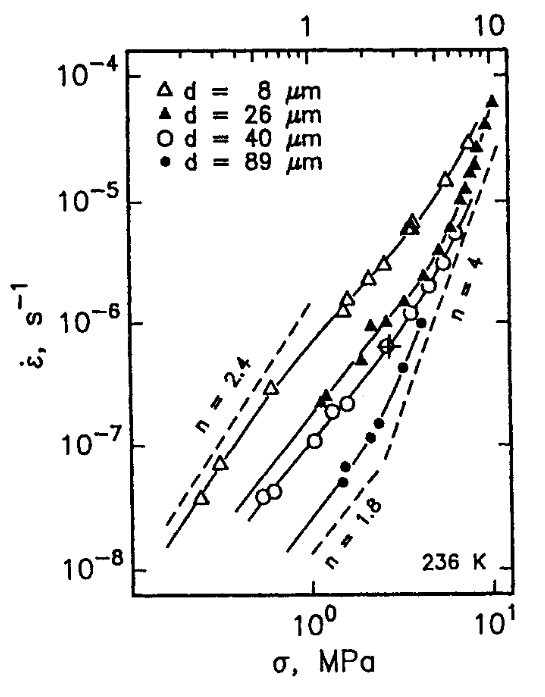

Supplement: Supplementary file 4 — Digitized data for published experiments and temperature measurements as a function of depth for the NEEM ice core. [file 41561_2025_1661_MOESM4_ESM.zip › Supplement_data2/Mechanical_data/Goldsby_Kohlstedt_1997/Capture1.PNG]

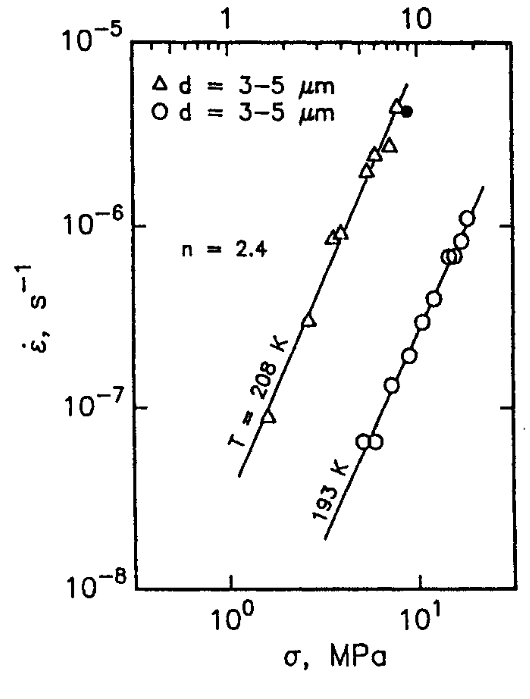

Supplement: Supplementary file 4 — Digitized data for published experiments and temperature measurements as a function of depth for the NEEM ice core. [file 41561_2025_1661_MOESM4_ESM.zip › Supplement_data2/Mechanical_data/Goldsby_Kohlstedt_1997/Capture2.PNG]
