# Supplementary material for: Flow laws for ice constrained by 70 years of laboratory experiments
Source: Nat Geosci. 2025 Mar 28;18(4):296–304. doi: 10.1038/s41561-025-01661-z (PMC11981940; doi:10.1038/s41561-025-01661-z)
Supplement: Supplementary file 5 — R code that includes comprehensive markdown notes detailing the Bayesian modelling process. [file 41561_2025_1661_MOESM5_ESM.zip › Supplement_code1/Bayesian_low_strain_one_component_GSS.pdf]

Bayesian modelling - low strain, one component (1C), GSS

Dr Sheng Fan

2024-07-22

1. Libraries and Data

The following libraries ae essential for the modelling and visualisation

- JAGS https://sourceforge.net/projects/mcmc-jags/
- R2jags https://cran.r-project.org/web/packages/R2jags/index.html
- lattice https://cran.r-project.org/web/packages/lattice/index.html
- ggplot2 https://cran.r-project.org/web/packages/ggplot2/index.html
- gridExtra https://cran.r-project.org/web/packages/gridExtra/index.html

```
# Import Libraries

library(R2jags)
library(lattice)
library(ggplot2)
library(gridExtra)

# Import data
file_path <- "D:\\Bayesian_modelling\\Input_data\\Supplement_S3_input_low_strain.csv"

data <- read.csv(file_path)

# Assignment of each column to a variable
Source <- data$Source
GS_ini_mean <- data$GS_ini_mean
GS_ini_upper <- data$GS_ini_upper
GS_ini_lower <- data$GS_ini_lower
rate_c <- data$rate_c
strain_c <- data$strain_c
stress_c <- data$stress_c
T_mean <- data$T_mean
T_upper <- data$T_upper
T_lower <- data$T_lower
exp_type <- data$exp_type
```

2. Bayesian Modelling

2.1 Set Bayesian modelling parameters and output dir

```
## Bayesian model parameters
# Iterations
n_iter <- 4000000
n_iter_str <- format (n_iter, scientific=FALSE)

# Burn-in
n_burnin <- 10000
n_burnin_str <- format (n_burnin, scientific=FALSE)

# Thinning
n_thin <- 20
n_thin_str <- format (n_thin, scientific=FALSE)

model <- "low_strain_single_component_GSS"
extension <- ".jags"
model_file <- paste0(model, extension)

## Output dir

strain_condition_input = "Low-strain"

law <- "GSS"

# Get the current date
date_time_str <- format(Sys.time(), "%Y-%m-%d-%H-%M-%S")

# Create the folder name by concatenating the date and variable name
sub_folder_name <- paste(date_time_str, strain_condition_input, law, n_iter_str, n_burnin_str, n_thin_s

# Create the folder under the master directory
new_dir <- file.path("D:\\Bayesian_modelling\\Results", sub_folder_name)

dir.create(new_dir)
```

2.2 Modelling

```
R <- 8.314*1e-3 #kJmol-1K-1

# number of observations
N <- length(stress_c)

rate_exp = log10(rate_c)

# model 0
data1 <- list("N"=N,
              "rate_exp" = rate_exp,
              "Stress"=stress_c,
```

```r
              "R" = R,
              "Init_GS"=GS_ini_mean,
              "Init_GS_upper"=GS_ini_upper,
              "Init_GS_lower"=GS_ini_lower,
              "T_mean"=T_mean,
              "T_upper"=T_upper,
              "T_lower"=T_lower)

vinits <- function(){
  list("n" = 4)
}

params <- c("n", "p", "Q","A_log")

# Start modelling
s1 <- Sys.time()
m0 = jags(data=data1, inits = vinits, parameters.to.save=params, n.chains = 3,
          n.iter = n_iter,n.burnin=n_burnin,n.thin=n_thin,
          model.file=model_file)
```

```
## module glm loaded
```

```
## Compiling model graph
##    Resolving undeclared variables
##    Allocating nodes
## Graph information:
##    Observed stochastic nodes: 305
##    Unobserved stochastic nodes: 614
##    Total graph size: 5532
##
## Initializing model
```

```r
s2 <- Sys.time()
s2-s1
```

```
## Time difference of 5.4049 hours
```

```r
m0
```

```
## Inference for Bugs model at "low_strain_single_component_GSS.jags", fit using jags,
##  3 chains, each with 4e+06 iterations (first 10000 discarded), n.thin = 20
##  n.sims = 598500 iterations saved
##          mu.vect sd.vect   2.5%    25%    50%    75%   97.5%  Rhat  n.eff
## A_log      3.307   0.277  2.766  3.120  3.306  3.492   3.852 1.001  56000
## Q         62.818   1.765 59.372 61.624 62.814 64.002  66.302 1.001  61000
## n          2.844   0.040  2.765  2.817  2.844  2.871   2.922 1.001 470000
## p          0.788   0.039  0.711  0.762  0.788  0.815   0.866 1.001 190000
## deviance  87.712   6.316 75.735 83.407 87.565 91.857 100.535 1.001 280000
##
## For each parameter, n.eff is a crude measure of effective sample size,
## and Rhat is the potential scale reduction factor (at convergence, Rhat=1).
##
```

```
## DIC info (using the rule, pD = var(deviance)/2)
## pD = 19.9 and DIC = 107.7
## DIC is an estimate of expected predictive error (lower deviance is better).
```

```r
m0.mcmc <- as.mcmc(m0)

# Document the summary of output
jags_output_path <- file.path(new_dir, "jags_output.txt")
sink(file = jags_output_path)

print(m0)
```

```
## Inference for Bugs model at "low_strain_single_component_GSS.jags", fit using jags,
##  3 chains, each with 4e+06 iterations (first 10000 discarded), n.thin = 20
##  n.sims = 598500 iterations saved
##          mu.vect sd.vect   2.5%    25%    50%    75%   97.5%  Rhat  n.eff
## A_log      3.307   0.277  2.766  3.120  3.306  3.492   3.852 1.001  56000
## Q         62.818   1.765 59.372 61.624 62.814 64.002  66.302 1.001  61000
## n          2.844   0.040  2.765  2.817  2.844  2.871   2.922 1.001 470000
## p          0.788   0.039  0.711  0.762  0.788  0.815   0.866 1.001 190000
## deviance  87.712   6.316 75.735 83.407 87.565 91.857 100.535 1.001 280000
##
## For each parameter, n.eff is a crude measure of effective sample size,
## and Rhat is the potential scale reduction factor (at convergence, Rhat=1).
##
## DIC info (using the rule, pD = var(deviance)/2)
## pD = 19.9 and DIC = 107.7
## DIC is an estimate of expected predictive error (lower deviance is better).
```

```r
summary(m0.mcmc)
```

```
##
## Iterations = 10001:3999981
## Thinning interval = 20
## Number of chains = 3
## Sample size per chain = 199500
##
## 1. Empirical mean and standard deviation for each variable,
##    plus standard error of the mean:
##
##             Mean      SD  Naive SE Time-series SE
## A_log     3.3066 0.27651 3.574e-04      2.496e-03
## deviance 87.7117 6.31609 8.164e-03      1.281e-02
## n         2.8437 0.04012 5.186e-05      6.397e-05
## p         0.7883 0.03945 5.099e-05      2.710e-04
## Q        62.8175 1.76484 2.281e-03      1.649e-02
##
## 2. Quantiles for each variable:
##
##             2.5%     25%     50%     75%   97.5%
## A_log     2.7662  3.1199  3.3062  3.4922   3.852
## deviance 75.7354 83.4065 87.5654 91.8570 100.535
## n         2.7651  2.8166  2.8437  2.8708   2.922
```

```
## p         0.7113  0.7616  0.7882  0.8148   0.866
## Q        59.3720 61.6242 62.8139 64.0016  66.302
```

```r
gelman.diag(m0.mcmc)
```

```
## Potential scale reduction factors:
##
##          Point est. Upper C.I.
## A_log             1          1
## deviance          1          1
## n                 1          1
## p                 1          1
## Q                 1          1
##
## Multivariate psrf
##
## 1
```

```r
sink(file = NULL)

# Save raw outputs
all_samples <- as.matrix (m0.mcmc[,])
all_samples_path <- file.path(new_dir, "all_samples.RDS")
save (all_samples, file=all_samples_path)

m0_file_path <- file.path(new_dir, "m0.RDS")
m0mc_file_path <- file.path(new_dir, "m0mc.RDS")

save(m0,file=m0_file_path)
save(m0.mcmc,file=m0mc_file_path)
```
