# Supplementary material for: Flow laws for ice constrained by 70 years of laboratory experiments
Source: Nat Geosci. 2025 Mar 28;18(4):296–304. doi: 10.1038/s41561-025-01661-z (PMC11981940; doi:10.1038/s41561-025-01661-z)
Supplement: Supplementary file 5 — R code that includes comprehensive markdown notes detailing the Bayesian modelling process. [file 41561_2025_1661_MOESM5_ESM.zip › Supplement_code1/Bayesian_low_strain_one_component_GSI.pdf]

Bayesian modelling - low strain, one component (1C), GSI

Dr Sheng Fan

2024-07-22

1. Libraries and Data

The following libraries ae essential for the modelling and visualisation

- JAGS https://sourceforge.net/projects/mcmc-jags/
- R2jags https://cran.r-project.org/web/packages/R2jags/index.html
- lattice https://cran.r-project.org/web/packages/lattice/index.html
- ggplot2 https://cran.r-project.org/web/packages/ggplot2/index.html
- gridExtra https://cran.r-project.org/web/packages/gridExtra/index.html

```r
# Import Libraries

library(R2jags)
library(lattice)
library(ggplot2)
library(gridExtra)

# Import data
file_path <- "D:\\Bayesian_modelling\\Input_data\\Supplement_S3_input_low_strain.csv"

data <- read.csv(file_path)

# Assignment of each column to a variable
Source <- data$Source
GS_ini_mean <- data$GS_ini_mean
GS_ini_upper <- data$GS_ini_upper
GS_ini_lower <- data$GS_ini_lower
rate_c <- data$rate_c
strain_c <- data$strain_c
stress_c <- data$stress_c
T_mean <- data$T_mean
T_upper <- data$T_upper
T_lower <- data$T_lower
exp_type <- data$exp_type
```

2. Bayesian Modelling

2.1 Set Bayesian modelling parameters and output dir

```
## Bayesian model parameters
# Iterations
n_iter <- 4000000
n_iter_str <- format (n_iter, scientific=FALSE)

# Burn-in
n_burnin <- 10000
n_burnin_str <- format (n_burnin, scientific=FALSE)

# Thinning
n_thin <- 20
n_thin_str <- format (n_thin, scientific=FALSE)

model <- "low_strain_single_component_GSI"
extension <- ".jags"
model_file <- paste0(model, extension)

## Output dir

strain_condition_input = "Low-strain"

law <- "GSI"

# Get the current date
date_time_str <- format(Sys.time(), "%Y-%m-%d-%H-%M-%S")

# Create the folder name by concatenating the date and variable name
sub_folder_name <- paste(date_time_str, strain_condition_input, law, n_iter_str, n_burnin_str, n_thin_s

# Create the folder under the master directory
new_dir <- file.path("D:\\Bayesian_modelling\\Results", sub_folder_name)

dir.create(new_dir)
```

2.2 Modelling

```
R <- 8.314*1e-3 #kJmol-1K-1

# number of observations
N <- length(stress_c)

rate_exp = log10(rate_c)

# model 0
data1 <- list("N"=N,
              "rate_exp" = rate_exp,
              "Stress"=stress_c,
```

```r
                "R" = R,
                "T_mean"=T_mean,
                "T_upper"=T_upper,
                "T_lower"=T_lower)

vinits <- function(){
  list("n" = 4)
}

params <- c("n", "Q","A_log")

# Start modelling
s1 <- Sys.time()
m0 = jags(data=data1, inits = vinits, parameters.to.save=params, n.chains = 3,
          n.iter = n_iter,n.burnin=n_burnin,n.thin=n_thin,
          model.file=model_file)
```

```
## module glm loaded
```

```
## Compiling model graph
##    Resolving undeclared variables
##    Allocating nodes
## Graph information:
##    Observed stochastic nodes: 305
##    Unobserved stochastic nodes: 308
##    Total graph size: 3888
##
## Initializing model
```

```r
s2 <- Sys.time()
s2-s1
```

```
## Time difference of 3.176176 hours
```

```r
m0
```

```
## Inference for Bugs model at "low_strain_single_component_GSI.jags", fit using jags,
##  3 chains, each with 4e+06 iterations (first 10000 discarded), n.thin = 20
##  n.sims = 598500 iterations saved
##          mu.vect sd.vect    2.5%     25%     50%     75%   97.5%  Rhat  n.eff
## A_log      0.393   0.233  -0.064   0.237   0.394   0.550   0.847 1.001  56000
## Q         36.045   1.142  33.798  35.278  36.046  36.815  38.277 1.001  59000
## n          3.072   0.038   2.998   3.047   3.072   3.097   3.146 1.001 420000
## deviance 500.582   2.876 496.165 498.603 500.150 502.082 507.457 1.001 600000
##
## For each parameter, n.eff is a crude measure of effective sample size,
## and Rhat is the potential scale reduction factor (at convergence, Rhat=1).
##
## DIC info (using the rule, pD = var(deviance)/2)
## pD = 4.1 and DIC = 504.7
## DIC is an estimate of expected predictive error (lower deviance is better).
```

```r
m0.mcmc <- as.mcmc(m0)

# Document the summary of output
jags_output_path <- file.path(new_dir, "jags_output.txt")
sink(file = jags_output_path)

print(m0)
```

```
## Inference for Bugs model at "low_strain_single_component_GSI.jags", fit using jags,
##  3 chains, each with 4e+06 iterations (first 10000 discarded), n.thin = 20
##  n.sims = 598500 iterations saved
##          mu.vect sd.vect    2.5%     25%     50%     75%   97.5%  Rhat  n.eff
## A_log      0.393   0.233  -0.064   0.237   0.394   0.550   0.847 1.001  56000
## Q         36.045   1.142  33.798  35.278  36.046  36.815  38.277 1.001  59000
## n          3.072   0.038   2.998   3.047   3.072   3.097   3.146 1.001 420000
## deviance 500.582   2.876 496.165 498.603 500.150 502.082 507.457 1.001 600000
##
## For each parameter, n.eff is a crude measure of effective sample size,
## and Rhat is the potential scale reduction factor (at convergence, Rhat=1).
##
## DIC info (using the rule, pD = var(deviance)/2)
## pD = 4.1 and DIC = 504.7
## DIC is an estimate of expected predictive error (lower deviance is better).
```

```r
summary(m0.mcmc)
```

```
##
## Iterations = 10001:3999981
## Thinning interval = 20
## Number of chains = 3
## Sample size per chain = 199500
##
## 1. Empirical mean and standard deviation for each variable,
##    plus standard error of the mean:
##
##              Mean      SD  Naive SE Time-series SE
## A_log      0.3933 0.23270 3.008e-04      1.555e-03
## deviance 500.5819 2.87584 3.717e-03      7.900e-03
## n          3.0720 0.03774 4.878e-05      9.923e-05
## Q         36.0454 1.14215 1.476e-03      7.634e-03
##
## 2. Quantiles for each variable:
##
##               2.5%      25%      50%      75%    97.5%
## A_log     -0.06393   0.2369   0.3935   0.5503   0.8475
## deviance 496.16547 498.6033 500.1496 502.0817 507.4571
## n          2.99786   3.0465   3.0720   3.0975   3.1459
## Q         33.79812  35.2784  36.0460  36.8153  38.2770
```

```r
gelman.diag(m0.mcmc)
```

```
## Potential scale reduction factors:
```

```
##
##          Point est. Upper C.I.
## A_log             1          1
## deviance          1          1
## n                 1          1
## Q                 1          1
##
## Multivariate psrf
##
## 1
```

```
sink(file = NULL)

# Save raw outputs
all_samples <- as.matrix (m0.mcmc[,])
all_samples_path <- file.path(new_dir, "all_samples.RDS")
save (all_samples, file=all_samples_path)

m0_file_path <- file.path(new_dir, "m0.RDS")
m0mc_file_path <- file.path(new_dir, "m0mc.RDS")

save(m0,file=m0_file_path)
save(m0.mcmc,file=m0mc_file_path)
```
