# Supplementary material for: Flow laws for ice constrained by 70 years of laboratory experiments
Source: Nat Geosci. 2025 Mar 28;18(4):296–304. doi: 10.1038/s41561-025-01661-z (PMC11981940; doi:10.1038/s41561-025-01661-z)
Supplement: Supplementary file 5 — R code that includes comprehensive markdown notes detailing the Bayesian modelling process. [file 41561_2025_1661_MOESM5_ESM.zip › Supplement_code1/Bayesian_low_strain_three_component.pdf]

Bayesian modelling - low strain, two component (3C): GSI+GSS1+GSS2

Dr Sheng Fan

2024-07-22

1. Libraries and Data

The following libraries ae essential for the modelling and visualisation

- JAGS https://sourceforge.net/projects/mcmc-jags/
- R2jags https://cran.r-project.org/web/packages/R2jags/index.html
- lattice https://cran.r-project.org/web/packages/lattice/index.html
- ggplot2 https://cran.r-project.org/web/packages/ggplot2/index.html
- gridExtra https://cran.r-project.org/web/packages/gridExtra/index.html

```
# Import Libraries

library(R2jags)
library(lattice)
library(ggplot2)
library(gridExtra)

# Import data
file_path <- "D:\\Bayesian_modelling\\Input_data\\Supplement_S3_input_low_strain.csv"

data <- read.csv(file_path)

# Assignment of each column to a variable
Source <- data$Source
GS_ini_mean <- data$GS_ini_mean
GS_ini_upper <- data$GS_ini_upper
GS_ini_lower <- data$GS_ini_lower
rate_c <- data$rate_c
strain_c <- data$strain_c
stress_c <- data$stress_c
T_mean <- data$T_mean
T_upper <- data$T_upper
T_lower <- data$T_lower
exp_type <- data$exp_type
```

2. Bayesian Modelling

2.1 Set Bayesian modelling parameters and output dir

```
## Bayesian model parameters
# Iterations
n_iter <- 4000000
n_iter_str <- format (n_iter, scientific=FALSE)

# Burn-in
n_burnin <- 10000
n_burnin_str <- format (n_burnin, scientific=FALSE)

# Thinning
n_thin <- 20
n_thin_str <- format (n_thin, scientific=FALSE)

model <- "low_strain_three_component"
extension <- ".jags"
model_file <- paste0(model, extension)

## Output dir

strain_condition_input = "Low-strain"

law <- "Three-component"

# Get the current date
date_time_str <- format(Sys.time(), "%Y-%m-%d-%H-%M-%S")

# Create the folder name by concatenating the date and variable name
sub_folder_name <- paste(date_time_str, strain_condition_input, law, n_iter_str, n_burnin_str, n_thin_s

# Create the folder under the master directory
new_dir <- file.path("D:\\Bayesian_modelling\\Results", sub_folder_name)

dir.create(new_dir)
```

2.2 Modelling

```
R <- 8.314*1e-3 #kJmol-1K-1

# number of observations
N <- length(stress_c)

rate_exp = log10(rate_c)

# model 0
data1 <- list("N"=N,
              "rate_exp" = rate_exp,
              "Stress"=stress_c,
```

```r
              "R" = R,
              "Init_GS"=GS_ini_mean,
              "Init_GS_upper"=GS_ini_upper,
              "Init_GS_lower"=GS_ini_lower,
              "T_mean"=T_mean,
              "T_upper"=T_upper,
              "T_lower"=T_lower)

vinits <- function(){
  list("n_dis" = 4)
}

params <- c("n_GBS_warm", "n_GBS_cold","n_dis", "p_warm", "p_cold", "Q_dis","Q_GBS_warm","Q_GBS_cold","A

# Start modelling
s1 <- Sys.time()
m0 = jags(data=data1, inits = vinits, parameters.to.save=params, n.chains = 3,
          n.iter = n_iter,n.burnin=n_burnin,n.thin=n_thin,
          model.file=model_file)
```

```
## module glm loaded
```

```
## Compiling model graph
##    Resolving undeclared variables
##    Allocating nodes
## Graph information:
##    Observed stochastic nodes: 305
##    Unobserved stochastic nodes: 621
##    Total graph size: 8317
##
## Initializing model
```

```r
s2 <- Sys.time()
s2-s1
```

```
## Time difference of 12.09502 hours
```

```r
m0
```

```
## Inference for Bugs model at "low_strain_three_component.jags", fit using jags,
##  3 chains, each with 4e+06 iterations (first 10000 discarded), n.thin = 20
##  n.sims = 598500 iterations saved
##                 mu.vect sd.vect    2.5%     25%     50%     75%   97.5%  Rhat
## A_GBS_cold_log   -0.946   0.737  -2.441  -1.429  -0.929  -0.443   0.455 1.001
## A_GBS_warm_log   22.820   2.006  19.077  21.426  22.750  24.154  26.909 1.001
## A_dis_log         5.020   1.008   2.935   4.372   5.057   5.704   6.905 1.001
## Q_GBS_cold       51.492   3.303  44.933  49.307  51.515  53.713  57.918 1.001
## Q_GBS_warm      182.296   9.997 162.613 175.523 182.305 189.125 201.741 1.001
## Q_dis            61.869   4.856  52.038  58.686  61.991  65.157  71.134 1.001
## n_GBS_cold        1.892   0.146   1.598   1.797   1.895   1.992   2.171 1.001
## n_GBS_warm        2.519   0.259   1.992   2.348   2.526   2.699   3.006 1.001
## n_dis             3.626   0.114   3.432   3.548   3.616   3.691   3.878 1.001
```

```
## p_cold           1.197   0.098   1.013   1.131   1.195   1.261   1.397 1.001
## p_warm           1.920   0.369   1.150   1.675   1.942   2.186   2.578 1.002
## deviance       -17.901   6.741 -30.210 -22.568 -18.229 -13.593  -3.740 1.001
##                  n.eff
## A_GBS_cold_log   43000
## A_GBS_warm_log    6700
## A_dis_log        12000
## Q_GBS_cold       15000
## Q_GBS_warm        4700
## Q_dis            16000
## n_GBS_cold       84000
## n_GBS_warm        5300
## n_dis            17000
## p_cold           12000
## p_warm            2600
## deviance        110000
##
## For each parameter, n.eff is a crude measure of effective sample size,
## and Rhat is the potential scale reduction factor (at convergence, Rhat=1).
##
## DIC info (using the rule, pD = var(deviance)/2)
## pD = 22.7 and DIC = 4.8
## DIC is an estimate of expected predictive error (lower deviance is better).
```

```r
m0.mcmc <- as.mcmc(m0)

# Document the summary of output
jags_output_path <- file.path(new_dir, "jags_output.txt")
sink(file = jags_output_path)

print(m0)
```

```
## Inference for Bugs model at "low_strain_three_component.jags", fit using jags,
##  3 chains, each with 4e+06 iterations (first 10000 discarded), n.thin = 20
##  n.sims = 598500 iterations saved
##                mu.vect sd.vect    2.5%     25%     50%     75%   97.5%  Rhat
## A_GBS_cold_log  -0.946   0.737  -2.441  -1.429  -0.929  -0.443   0.455 1.001
## A_GBS_warm_log  22.820   2.006  19.077  21.426  22.750  24.154  26.909 1.001
## A_dis_log        5.020   1.008   2.935   4.372   5.057   5.704   6.905 1.001
## Q_GBS_cold      51.492   3.303  44.933  49.307  51.515  53.713  57.918 1.001
## Q_GBS_warm     182.296   9.997 162.613 175.523 182.305 189.125 201.741 1.001
## Q_dis           61.869   4.856  52.038  58.686  61.991  65.157  71.134 1.001
## n_GBS_cold       1.892   0.146   1.598   1.797   1.895   1.992   2.171 1.001
## n_GBS_warm       2.519   0.259   1.992   2.348   2.526   2.699   3.006 1.001
## n_dis            3.626   0.114   3.432   3.548   3.616   3.691   3.878 1.001
## p_cold           1.197   0.098   1.013   1.131   1.195   1.261   1.397 1.001
## p_warm           1.920   0.369   1.150   1.675   1.942   2.186   2.578 1.002
## deviance       -17.901   6.741 -30.210 -22.568 -18.229 -13.593  -3.740 1.001
##                  n.eff
## A_GBS_cold_log   43000
## A_GBS_warm_log    6700
## A_dis_log        12000
## Q_GBS_cold       15000
## Q_GBS_warm        4700
```

```
## Q_dis           16000
## n_GBS_cold      84000
## n_GBS_warm       5300
## n_dis           17000
## p_cold          12000
## p_warm           2600
## deviance       110000
##
## For each parameter, n.eff is a crude measure of effective sample size,
## and Rhat is the potential scale reduction factor (at convergence, Rhat=1).
##
## DIC info (using the rule, pD = var(deviance)/2)
## pD = 22.7 and DIC = 4.8
## DIC is an estimate of expected predictive error (lower deviance is better).
```

```r
summary(m0.mcmc)
```

```
##
## Iterations = 10001:3999981
## Thinning interval = 20
## Number of chains = 3
## Sample size per chain = 199500
##
## 1. Empirical mean and standard deviation for each variable,
##    plus standard error of the mean:
##
##                    Mean      SD  Naive SE Time-series SE
## A_dis_log        5.0195 1.00754 0.0013024      0.0169870
## A_GBS_cold_log  -0.9457 0.73696 0.0009526      0.0065073
## A_GBS_warm_log  22.8202 2.00623 0.0025933      0.0323339
## deviance       -17.9010 6.74083 0.0087133      0.0256165
## n_dis            3.6255 0.11356 0.0001468      0.0005556
## n_GBS_cold       1.8925 0.14589 0.0001886      0.0009218
## n_GBS_warm       2.5194 0.25948 0.0003354      0.0023804
## p_cold           1.1973 0.09778 0.0001264      0.0005108
## p_warm           1.9203 0.36897 0.0004769      0.0041583
## Q_dis           61.8687 4.85552 0.0062763      0.0811832
## Q_GBS_cold      51.4921 3.30286 0.0042693      0.0294390
## Q_GBS_warm     182.2958 9.99706 0.0129223      0.1610838
##
## 2. Quantiles for each variable:
##
##                   2.5%     25%      50%     75%    97.5%
## A_dis_log        2.935   4.372   5.0565   5.704   6.9053
## A_GBS_cold_log  -2.441  -1.429  -0.9294  -0.443   0.4553
## A_GBS_warm_log  19.077  21.426  22.7502  24.154  26.9086
## deviance       -30.210 -22.568 -18.2289 -13.593  -3.7404
## n_dis            3.432   3.548   3.6156   3.691   3.8782
## n_GBS_cold       1.598   1.797   1.8954   1.992   2.1708
## n_GBS_warm       1.992   2.348   2.5261   2.699   3.0058
## p_cold           1.013   1.131   1.1948   1.261   1.3971
## p_warm           1.150   1.675   1.9421   2.186   2.5784
## Q_dis           52.038  58.686  61.9906  65.157  71.1335
## Q_GBS_cold      44.933  49.307  51.5150  53.713  57.9184
```

```
## Q_GBS_warm     162.613 175.523 182.3045 189.125 201.7409
```

```r
gelman.diag(m0.mcmc)
```

```
## Potential scale reduction factors:
##
##                Point est. Upper C.I.
## A_dis_log               1       1.00
## A_GBS_cold_log          1       1.00
## A_GBS_warm_log          1       1.00
## deviance                1       1.00
## n_dis                   1       1.00
## n_GBS_cold              1       1.00
## n_GBS_warm              1       1.00
## p_cold                  1       1.00
## p_warm                  1       1.01
## Q_dis                   1       1.00
## Q_GBS_cold              1       1.00
## Q_GBS_warm              1       1.00
##
## Multivariate psrf
##
## 1
```

```r
sink(file = NULL)

# Save raw outputs
all_samples <- as.matrix (m0.mcmc[,])
all_samples_path <- file.path(new_dir, "all_samples.RDS")
save (all_samples, file=all_samples_path)

m0_file_path <- file.path(new_dir, "m0.RDS")
m0mc_file_path <- file.path(new_dir, "m0mc.RDS")

save(m0,file=m0_file_path)
save(m0.mcmc,file=m0mc_file_path)
```
